# Inverse game theory characterizes frequency-dependent selection driven by karyotypic diversity in triple-negative breast cancer

**DOI:** 10.1371/journal.pcbi.1013897

**Published:** 2026-03-10

**Authors:** Thomas Veith, Richard J. Beck, Joel S. Brown, Noemi Andor

**Affiliations:** 1 Integrated Mathematical Oncology, H. Lee Moffitt Cancer Center, Tampa, Florida, United States of America; 2 Department of Molecular Biosciences, University of South Florida, Tampa, Florida, United States of America; 3 Department of Oncologic Sciences, University of South Florida, Tampa, Florida, United States of America; University of Leeds, UNITED KINGDOM OF GREAT BRITAIN AND NORTHERN IRELAND

## Abstract

Chromosomal instability, characterized by pervasive copy number alterations (CNAs), significantly contributes to cancer progression and therapeutic resistance. CNAs drive intratumoral genetic heterogeneity, creating distinct subpopulations whose interactions shape tumor evolution through frequency-dependent selection. Here, we introduce ECO-K (Ecological-Karyotypes), an inverse game theory framework that quantifies frequency-dependent interaction coefficients among karyotypically defined subpopulations under the assumption that their fitness is frequency-dependent. Applying this approach to serially-passaged, triple-negative breast cancer cell lines and patient-derived xenografts (PDXs), we estimated interaction matrices consistent with the observed time-series dynamics. In one PDX lineage, the inferred matrices consistently assigned large interaction coefficients to a subpopulation characterized by chromosome 1 loss and chromosome 14p gain, suggesting it may act as an ecological hub within the frequency-dependent model. Our framework provides testable predictions of intratumoral ecological dynamics, highlighting opportunities to strategically target key subpopulations to disrupt tumor evolution.

## Author summary

Cancer evolves rapidly because tumor cells continuously change their genetic makeup, particularly through alterations in chromosome numbers via a process called chromosomal instability. This genetic variation allows multiple distinct cell populations to emerge within a tumor, each competing or cooperating with others. Understanding these interactions could reveal new ways to disrupt tumor growth. We introduce a computational approach called ECO-K, inspired by evolutionary game theory. ECO-K analyzes genetic data from single cancer cells collected over time to infer how these cell populations interact. We applied this method to breast cancer cells grown in the lab and tumors grown in mouse models. Our results identified specific cancer cell populations acting as critical “hubs”, influencing the survival and proliferation of neighboring cells. By pinpointing these influential populations, ECO-K reveals potential vulnerabilities in the tumor’s internal ecological network.

## 1 Introduction

The evolutionary process that unfolds during tumor progression often renders failure in the clinic a *fait accompli* as natural selection promotes the expansion of therapeutically resistant subpopulations [1,2]. Chromosomal instability, a hallmark of cancer associated with advanced disease and poor outcomes [3], is a powerful evolutionary process that rapidly generates genetic diversity. Chromosomal instability results in ongoing copy number alterations which are pervasive across cancer types [4], affecting larger segments of the cancer genome than any other alteration [5]. While CNAs are known to alter a cancer cell’s fitness [4], response to treatment [6], and immunogenecity [4], it remains unclear how distinct karyotypes (SPs) differ in phenotype or whether they engage in frequency-dependent interactions that influence tumor evolution.

To conceptualize these cellular interactions, we can turn to evolutionary game theory (EGT), a framework that models how the success of an individual strategy depends on the strategies of others in the population. The core concept is frequency-dependent selection, where a cell’s fitness is dynamic, changing based on the relative abundance of other cell types in the microenvironment. A simple analogy is the game of rock-paper-scissors. The success of “rock” is entirely dependent on the frequency of “scissors” (which it beats) and “paper” (which it loses to). In this game, the outcomes of every possible interaction can be summarized in the payoff matrix, which defines the rules of the game. In cancer, this means one karyotype might outcompete another when rare, but be at a disadvantage when common, leading to complex, non-linear growth dynamics.

A core principle in EGT is strategic equivalence: different payoff matrices can generate identical dynamics because only relative fitness differences matter, not absolute payoff values. For instance, adding a constant to all payoffs leaves competitive outcomes unchanged. This is crucial for our inverse approach, where the goal is not to recover a single “true” matrix but rather any representative matrix that captures the frequency-dependent interactions within the tumor. From these interactions, we can infer long-term outcomes such as an evolutionarily stable strategy (ESS), a population composition resistant to invasion by rare mutants. An ESS may be pure (all cells identical) or mixed, and in tumors could correspond to uniformly sensitive, uniformly resistant, or coexisting resistant and sensitive SPs.

EGT has provided valuable insights into many hallmarks of cancer, including tumor-immune interactions [7], angiogenesis [8], invasion [9–11], evasion of apoptosis [12], and clonal evolution [13,14]. However, a limitation of these approaches is that they either assumed *a priori* knowledge of the rules which govern these interactions or applied their method to co-culture systems where the heterogeneity was introduced by the experimentalists themselves, as opposed to applying their method to preexisting heterogeneous populations. To address this gap, we introduced an inverse game theory (IGT) approach that systematically inferred the payoff matrix directly from empirical, longitudinal data of serially-passaged triple negative breast cancer (TNBC) PDX tumors in cell cultures. By applying this method to already co-existing, karyotypically distinct SPs, we quantitatively defined the rules of their interactions. This allowed us to systematically evaluate frequency-dependent selection within the tumors, revealing how the presence or absence of specific SPs fundamentally altered the competitive landscape and enhanced or impaired overall tumor growth.

## 2 Materials and methods

In this section, we describe the experimental foundations and computational framework for the estimation of frequency-dependent interaction coefficients among co-existing karyotypes (further referred to as SPs). To start, we provide a summary of the longitudinal single-cell whole genome sequencing (scWGS) datasets generated from serially-passaged cell lines and PDX models [15], including details of sampling and treatment exposures. We then outline how we took those single-cell genomes and aggregated their copy number profiles at the chromosome-arm level, clustered them into karyotype-defined SPs, and grouped them into biological replicates, forming the basis for temporal SP frequency data. Following this, we describe how we calculated correlations between SP frequencies and growth rates to identify payoff matrix entries which were candidates for non-zero parameterization. Subsequent sections detail the optimization routine for estimating payoff entries under replicator dynamics, model selection via iterative BIC calculations, and the use of parametric bootstrapping to assess robustness. Finally, we describe how we validated our approach on synthetic datasets designed to test inference accuracy across varying noise and complexity regimes.

### 2.1 Longitudinal single-cell whole genome sequencing data

In their work [15], Salehi et al. passaged the TNBC cell line 184-hTERT to characterize subpopulation dynamics. They employed CRISPR-CAS9 to derive a p53 knock-out (KO) version of the 184-hTERT cell line from the parental p53 wild-type (WT) cell line. The parental (WT) line was cultured for 60 passages and sampled four times to infer copy number status via scWGS (1599 cells sequenced on average, ranging from 1135-2061, 6395 total). Similarly, the KO cell line was cultured for 60 passages, with a replicate KO dish established at passage 20, which resulted in two KO lineages which were sampled for scWGS a total of six times, establishing single-cell genomes for 1200 cells per passage (range of 728–1738 cells, 16795 cells total). None of the *in-vitro* experiments involved cisplatin treatment.

**Fig 1:**
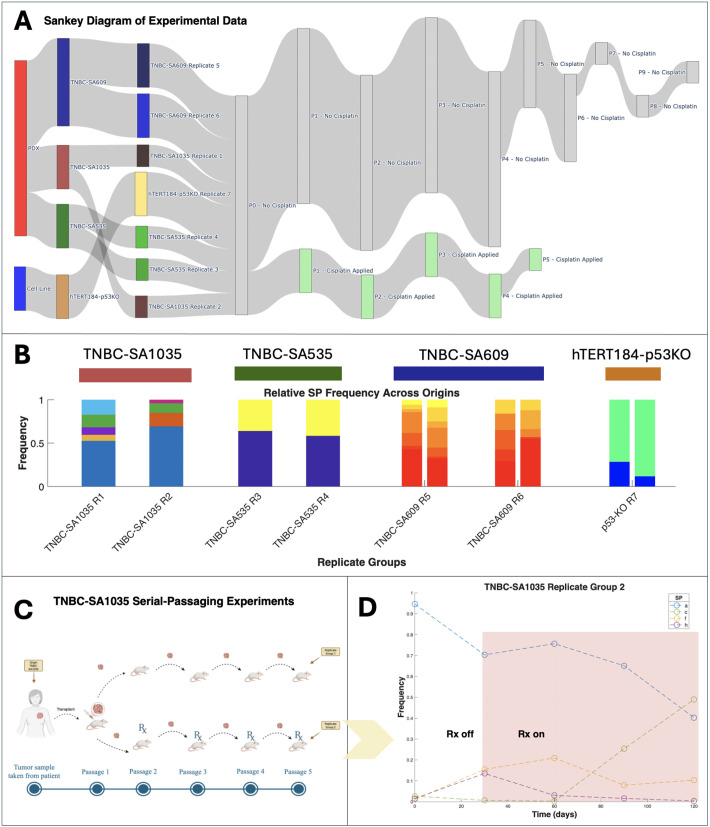
Overview of the serial-passaging experiments and example subpopulation dynamics in the SA1035-TNBC treatment arm. **(A)** Sankey diagram showing data origin (cell line or PDX), replicate groups, and cisplatin treatment schedules. **(B)** Relative SP frequencies across origins and replicate groups, with colors indicating SP identities unique to each replicate group. **(C)** Schematic of serial-passaging in the TNBC-SA1035 PDX, illustrating tumor passaging under treatment and no treatment arms. Created in BioRender. Veith, T. (2026) https://BioRender.com/yq988eq. **(D)** Example SP dynamics from TNBC-SA1035 Replicate Group 2, highlighting SP frequency shifts over time. Shaded region indicates cisplatin administration.

Expanding their study [15] to *in-vivo* models, the researchers established one HER2+ and three TNBC PDX tumor mouse models. From these four PDX tumors, a total of 27 lineages were established. Among these, six serially-passaged PDX tumors were never exposed to treatment, while 18 were exposed to cisplatin in at least one passage. The PDX tumors were passaged between four and seven times over a range of 353–927 days, with single-cell genomes established at each passage for an average of 1146 cells originating from TNBC PDX mouse models (range of 466–1845 cells, 95954 total), and 890 cells from the HER2 + PDX (ranging from 497-1831 for a total of 8009 cells). For all downstream analysis, we only considered those lineages which had no intermittent treatment (Fig 1).

### 2.2 Defining subpopulations and replicate groups

We took the single-cell copy number calls generated by Salehi et al. (2021) and aggregated them to chromosome arm–level profiles by annotating genomic bins and computing modal arm-level copy numbers (Sect 2.2.1). SPs were then identified by applying k-means clustering (k∈{2,…,10}) with Silhouette scores used to determine the optimal number of clusters (Sect 2.2.2). Finally, replicate groups were defined based on lineage origin and treatment exposure, and for each replicate group a subpopulation frequency matrix was computed to capture temporal dynamics of SP composition across passages (Sect 2.2.3).

#### 2.2.1 Copy number calling and chromosome arm aggregation.

We first processed single-cell copy number data to generate chromosome arm level copy number profiles. For each input file (a segment-by-cell copy number matrix was generated as a compressed CSV), the genomic bins were annotated with their corresponding chromosome and band information using a loci file. For each chromosome arm per cell, the copy number was taken as the modal value across all segments mapping to that arm. The resulting cell by arm-level copy number matrix was saved for subsequent clustering.

#### 2.2.2 Subpopulation assignment via k-means clustering.

For each patient-derived xenograft or cell line, we aggregated the arm-level copy number matrices from all available single-cell datasets. Subpopulation (SP) identities were then determined by applying k-means clustering to the aggregated matrix. Specifically, for a given k (with k∈{2,…,10}), clustering was performed with 25 random starts, and the average Silhouette score computed. The optimal number of SPs n was selected as:


$$n=\arg\max_{k\in 2,\ldots ,10}s({𝒦}_{k})$$ (1)

where $s({𝒦}_{k})$ is the average Silhouette score for the clustering with k centroids. Each cell is thereby assigned to a subpopulation (denoted $\kappa_{i}$, i=1,…,n). We selected k-means clustering for its interpretability and suitability for discrete subpopulation (SP) identification in copy number space (S1 Text, Sect 5.7). K-means clustering was performed using the base package ‘stats’ in R (4.4.1) with Silhouette scores calculated using the ‘cluster’ (2.4.1) package.

#### 2.2.3 Computing subpopulation-frequency matrices and replicate grouping.

Two serially-passaged lineages were considered members of the same biological replicate group if and only if they originated from the same PDX or cell line and had been exposed to cisplatin for the same number passages. We implemented the replicate structure to explore the robustness of our inverse search, as finding one matrix that described the interactions of more than one dataset (containing the same karyotype-defined SPs) increases the confidence that interactions between those karyotypes are real.

Using available metadata, each cell was annotated with its passage (timepoint) and an experimental label that reflects treatment exposure. Although SPs were defined over all cells from an origin, cells were further grouped by timepoint. For each timepoint t (or passage) within a replicate group, we computed a SP-frequency matrix ${𝐗}_{r}\in {\mathbb{R}}_{+}^{n\times \tau }$, where each entry was defined as:


$$x_{it}=\frac{\left|{𝒞}_{rt}\cap \kappa_{i}\right|}{\left|{𝒞}_{rt}\right|}.$$ (2)

Here, ${𝒞}_{rt}$ represents the set of cells in replicate r at timepoint t. Thus, $x_{it}$ is the fraction of cells ${𝒞}_{rt}$ that belong to the $i^{th}$ subpopulation, $\kappa_{i}$.

### 2.3 Identifying candidate interactions

From the SP frequency matrices, we filtered for candidate interactions by identifying pairs where the growth rate of one subpopulation was significantly correlated with the frequency of another (see S1 Text, Sect 5.1).

### 2.4 Optimization routine

To capture the dynamics of SP interactions and selection pressures, we modeled frequency-dependent selection using the replicator equation. This equation described the change in frequency of each SP over time, incorporating the fitness of each SP and the population-average fitness. For n SPs, the replicator equation is given by:


$${\dot{x}}_{\left(it\right)}=x_{\left(it\right)}[f_{\left(it\right)}-\bar{f_{t}}]$$ (3)

where $x_{it}$ is the frequency of the $i^{\text{th}}$ SP as defined in Eq (2), $f_{it}$ gives that SP’s fitness, and $\bar{f_{t}}$ is the population-average fitness. The fitness of a given SP was defined as:


$$f_{it}=\sum_{j=1}^{n}x_{jt}M_{ij}$$ (4)

where M is the payoff matrix where an entry $M_{ij}$ is the payoff to an individual of SP i when interacting with an individual of SP j. Consequently, the population-average fitness $\bar{f_{t}}$ is the weighted average of all individual fitness values:


$$\bar{f_{t}}=\sum_{i=1}^{n}x_{it}f_{it}$$ (5)

Some interactions between SPs may have no fitness consequences. In that case the payoff matrix entry representing their interaction would be set to zero. Non-zero payoff matrix entries were inferred via the methods described in Sect 2.3.

The final payoff matrix M was determined using a hybrid routine that combined model selection and parameter estimation. The overall structure of the matrix (i.e., the set of non-zero interaction coefficients) was estimated using a beam search algorithm. This search performs a guided, backward-elimination of parameters, starting from a full model. At each iteration, it evaluates simplified models created by setting a single interaction coefficient to zero. To prevent a greedy search, a beam width of 3 was used to maintain and expand upon the most promising model structures at each step.

The fitness of each candidate model structure was evaluated by its Bayesian Information Criterion (BIC) (see Sect 2.5). This inner optimization was performed using the sequential quadratic programming (SQP) algorithm, as implemented in MATLAB’s fmincon function (MATLAB R2025a). To ensure a robust estimation and avoid local minima, each SQP optimization was launched from 80 random starting points using the MultiStart framework. The final model selected was the one with the lowest BIC found across the entire beam search procedure.

Parameters were estimated by minimizing the negative log-likelihood assuming Gaussian residuals (see S1 Text, Sect 5.2).

The optimization routine was performed for multiple replicates simultaneously. Let $\Omega :=\{X_{r1},X_{r2},\ldots$} be the set of replicates within a replicate group, with entries as defined in Eq (2). To reduce the incidence of spurious subpopulations with noisy frequency dynamics, we excluded from all downstream analysis a given subpopulation which never exceeds 10% frequency ∀t∈T in any replicate. Suppose one such SP exists, then n:=n−1. Note that this leads to the payoff matrix M having identical dimensions across all replicates of a given group during each iteration of the optimization routine.

### 2.5 Parsimonious parametrization based on iterative BIC calculation

We used the above optimization routine to select between models of different complexity as follows. We calculated the BIC and corrected Akaike Information Criterion (AICc) across all replicates within a given group. These were given by:


BIC(Ω)=2ℒ+ρlnτ (6)

$$\text{AICc}(\Omega )=2ℒ+2\rho +\frac{2\rho (\rho +1)}{\tau -\rho -1}$$ (7)

where ℒ is the negative log-likelihood calculated in Sect 2.4, ρ is the total number of estimated parameters (the non-zero entries in the payoff matrix M) and τ is the total number of passages across all replicates.

During each iteration, the current set of non-zero payoff matrix entries was re-estimated (see Methods Sect 2.4), and the corresponding BIC and AICc calculated. Each non-zero entry was then systematically considered for removal, one at a time, by setting it to zero and re-optimizing the model. For each candidate removal, the fit was re-evaluated, and the BIC and AICc recalculated. The removal that yielded the most favorable change was adopted, and the process repeated. To guard against local optima, entries that did not individually improve model fit were also tested in combination with other removals. This combinatorial search continued iteratively until only two non-zero entries remained. Lastly, the best combination of non-zero and zero entries (yielding the best BIC) across the entire search was retained. BIC was used as the primary model selection criterion because it applies a stronger penalty for additional parameters compared to AIC/AICc, which helps prevent overfitting, particularly in settings where the number of potential payoff matrix entries can be large relative to the number of sampling timepoints. For replicate groups with more than 2 SPs, allowing both ECO-K and FitClone to be applied, we computed AICc and BIC for each model using their respective likelihood functions. For model comparison, we required concordant improvement in both criteria: ECO-K was judged superior only if both AICc and BIC were lower than those of FitClone; otherwise FitClone was considered to provide the better fit (see Table 1).

**Table 1:** Comparison of ECO-K vs FitClone performance across various TNBC lineages. The table summarizes the root mean squared error (RMSE), AICc, and BIC for both ECO-K and FitClone models. Each row represents an individual sample with column entries to denote replicate group (RG), the number of SPs in that replicate group, the number of non-zero interaction coefficients estimated by ECO-K, the number of those coefficients deemed significant during parametric bootstrapping, the mean absolute matrix entry value (payoff), the number of timepoints at which single-cell genomes were established, and at how many of those timepoints the sample had been exposed to cisplatin. Overall, ECO-K provided better fits (lower AICc and BIC) in two datasets, where FitClone was superior in two datasets. The remaining four datasets were unable to be analyzed by FitClone as it requires more than two SPs.

Sample	RG	SPs	Interactions	Significant	Mean | Payoff |	Timepoints	Rx Timepoints	RMSE	AICc	BIC	fitClone RMSE	fitClone AICc	fitClone BIC
TNBC-SA1035	1	5	7	7	0.51	5	0	0.02	-68.09	-70.82	0.00	**-84.84**	**-88.36**
TNBC-SA1035	2	4	4	4	0.52	5	4	0.03	-50.58	-52.15	0.03	**-62.05**	**-64.78**
TNBC-SA535	3	2	3	3	0.94	6	5	0.11	342.01	329.38	–	–	–
TNBC-SA535	4	2	3	3	0.16	5	0	0.02	-2.63	-27.80	–	–	–
TNBC-SA609	5	7	13	11	0.46	7	0	0.05	**662.09**	**551.58**	0.20	730.60	729.89
TNBC-SA609	5	7	13	11	0.46	10	0	0.05	**662.09**	**551.58**	0.11	730.60	734.53
TNBC-SA609	6	6	9	8	0.41	5	0	0.05	**84.58**	**87.31**	0.21	265.31	261.01
TNBC-SA609	6	6	9	8	0.41	5	0	0.05	**84.58**	**87.31**	0.07	265.31	261.01
184-hTERT p53${\;}^{-}{/}^{-}$	7	2	3	3	0.66	7	0	0.07	185.46	184.97	–	–	–
184-hTERT p53${\;}^{-}{/}^{-}$	7	2	3	3	0.66	7	0	0.05	185.46	184.97	–	–	–

### 2.6 Confidence interval estimation via parametric bootstrapping

To ensure the reliability of matrix entry estimates, we employed a subsampling-based method. For each 𝐗∈Ω, we generated β=1000 subsampled data sets $\left\{{𝐗}_{1}^{*},{𝐗}_{2}^{*},\ldots ,{𝐗}_{\beta }^{*}\right\}$ by randomly selecting unique timepoints (i.e., sampling without replacement) from the solution to Eq (3) using the payoff matrix 𝐌 found via ECO-K. Specifically, each subsample ${𝐗}_{b}^{*}$ was formed by drawing a random subset of timepoints and the corresponding solution trajectories from the replicator equation. The number of timepoints used in the subsampled datasets was equal to the original number of timepoints available for that replicate, minus one.

For each subsampled data set ${𝐗}_{b}^{*}$, we performed the optimization routine as described in Sect 2.4, which resulted in subsampled payoff matrices ${\hat{𝐌}}_{b}$ for b=(1,2,…,β). After processing all subsampled data sets, we analyzed the resulting payoff matrix estimates $\left\{{\hat{𝐌}}_{1},{\hat{𝐌}}_{2},\ldots ,{\hat{𝐌}}_{\beta }\right\}$ and calculated the means, standard errors, p-values, and confidence intervals of these estimates to assess their variability and statistical significance.

In particular, the mean and standard error of the estimated payoff matrix entries were given by:


$${\hat{𝐌}}_{\text{mean}}=\frac{1}{\beta }\sum_{b=1}^{\beta }{\hat{𝐌}}_{b},$$

$${\hat{\sigma }}_{\hat{𝐌}}=\sqrt{\frac{1}{\beta -1}\sum_{b=1}^{\beta }({\hat{𝐌}}_{b}-{\hat{𝐌}}_{\text{mean}}{)}^{2}}.$$

The 95% confidence intervals can be calculated using the percentiles of the subsampled distribution or using the standard error:


$${\text{CI}}_{95\%}=({\hat{𝐌}}_{\text{mean}}-1.96\cdot {\hat{\sigma }}_{\hat{𝐌}},\;{\hat{𝐌}}_{\text{mean}}+1.96\cdot {\hat{\sigma }}_{\hat{𝐌}}).$$

We tested the null hypothesis that a matrix entry was effectively zero ($H_{0}:{\hat{𝐌}}_{\text{mean}}=0$. This test was performed by calculating a 𝐭-statistic (using the MATLAB R2025a *ttest2* function) for the mean of the bootstrapped estimates, ${\hat{𝐌}}_{\text{mean}}$, against a value of zero, using the parametric bootstrap standard error ${\hat{\sigma }}_{\hat{𝐌}}$. Bootstrap parameters which returned a p-value < 0.05 were considered significantly different than zero.

### 2.7 Analysis of resulting payoff matrices

#### 2.7.1 Replicator phase diagrams.

To visualize the structure of interactions encoded in payoff matrices, we implemented a Python (3.11.3) script that converts each matrix into a directed network representation (Fig 4C). Nodes correspond to individual SPs (strategies), and directed edges represent non-zero payoff values, with their color and width indicating the sign and magnitude of the interaction coefficient. Positive payoffs were plotted as blue edges, negative payoffs as red edges, and edge thickness was scaled proportionally to the absolute payoff magnitude. Self-interactions were explicitly included when the diagonal entries of the payoff matrix were non-zero, rendered as self-loops with curved arcs. The script utilized the “networkx” (3.5) library to construct a directed graph from the payoff matrix, and nodes were positioned using a circular layout to emphasize symmetry among SPs.

#### 2.7.2 Total velocity magnitude ternary plots.

We utilized the IsoMaTrix package [16] for MATLAB R2025a to generate ternary phase diagrams based on payoff matrices using the “isomatrix-velocity” function (Fig 4D). The function accepts a 3×3 payoff matrix and computes the corresponding replicator dynamics across the simplex defined by all possible frequency combinations of three competing SPs. A uniform grid of initial conditions was constructed over the simplex, and the velocity field was evaluated at each point using the replicator equation (Eq 3). The script projects this vector field onto barycentric coordinates to render the dynamics on a triangular domain. The total velocity magnitude was computed at each grid point and visualized as a heat map using a continuous colormap, with higher intensity regions corresponding to faster evolutionary change. In systems with more than three SPs, ternary plots were generated by subsetting the payoff matrix to focus on a specific triplet of SPs.

#### 2.7.3 Calculating population average fitness of varying SP compositions.

To evaluate how population fitness depends on different combinations of SPs, we systematically re-simulated the replicator dynamics for every subset of SPs of size two or greater. For each subset, we extracted the relevant rows and columns from the full payoff matrix to create a smaller submatrix that only included the selected SPs. Initial conditions for these reduced systems were set by taking the average frequencies observed across the first three timepoints of the full dataset, restricted to the SPs in the subset and renormalized to sum to one.

The replicator dynamics were then simulated over the full experimental time window using the same solver and time settings as in the main analysis. At each simulated timepoint, we recorded the population’s average fitness ($\bar{f_{t}}$). For every subset, we retained the mean fitness across the time course. These results allowed us to plot fitness dynamics for specific SP combinations and to compare average fitness across all possible SP subsets (Fig 4F).

### 2.8 Synthetic dataset generation

To test the robustness of our method, we generated 1000 artificial datasets with varying numbers of SPs and noise levels. For each dataset, we first randomly selected the number of SPs (n, where 2≤n≤5) and assigned initial SP frequencies $X_{0}=\{X_{i0},X_{j0},\ldots ,X_{n0}\}$ drawn from a uniform distribution, normalized such that they sum to 1.

Next, we generated a random payoff matrix M by drawing entries uniformly from the interval [−1,1]. To restrict interaction complexity while still allowing for a sufficient number of nonzero payoff matrix entries, we kept the top 2n−1 matrix entries by absolute value and set the rest to zero. We then solved the replicator dynamics (Eq 3) from t=0 to t=50 with these initial conditions. To assess how accurately we could infer the original payoff matrix, we used the following two performance metrics:

- **Rank Correlation:** $\rho (M,M^{\prime })$, the Spearman’s rank correlation coefficient between the vectorized entries of the true matrix M and the inferred matrix $M^{\prime }$. This measures the model’s ability to recover the correct relative ordering of the payoff entries, independent of their absolute values (Figs 3A–3B, S2). The existence of an ESS and the qualitative features of the fitness landscape were determined by the relative ordering of payoffs. A high rank correlation indicates that the inferred payoff matrix preserves these strategic properties.
- **Dynamic Range:** $\text{range}(M^{\prime })=\max(M^{\prime })-\min(M^{\prime })$, the difference between the maximum and minimum entries in the inferred matrix. The Spearman correlation coefficient between $\text{range}(M^{\prime })$ and the true range, range(M), was used to evaluate how well the model captures the strength of selection, dictated by the magnitude of payoff differences (Figs 3C–3D, S3). An accurately inferred range indicates that the model correctly predicts the rate at which strategy frequencies will change under replicator dynamics.

## 3 Results

### 3.1 Inverse game theory estimates karyotype interaction coefficients from interference patterns

**Fig 2:**
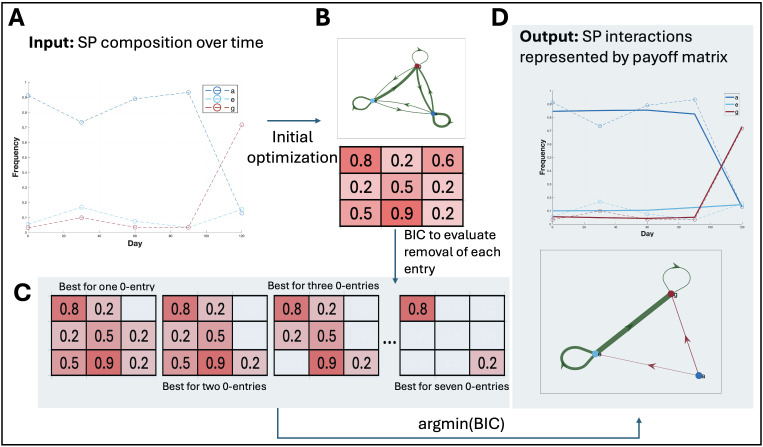
Overview of the optimization routine used in our method. The flowchart depicts the step-by-step process of optimizing the payoff matrix for SP interactions using ECO-K. The routine begins by initializing the interactions and setting up the optimization problem. An initial optimization was performed, and the BIC was calculated with all interactions. The method then evaluates the removal of matrix entries to determine if this improves the BIC score. All indices in the matrix were tested for removal, but only the one that gave the lowest BIC score was retained. This routine was designed to ensure the most parsimonious set of interactions was used to capture the subpopulation dynamics.

We developed a comprehensive framework to model and quantify frequency-dependent interactions between SPs in genomically resolved time-series data (Fig 2). Briefly, we first identified candidate SP pairs for inclusion in the model by testing whether the growth rate of one SP correlated with the frequency of the other SP [13]. We then optimized a payoff matrix (where each entry reflects the interaction strength for the corresponding SP pair) by minimizing a negative log-likelihood function under a multi-start strategy (Eq (12)). After this optimization, we iteratively removed individual entries from the payoff matrix if their removal lowered the BIC, thereby pruning insignificant matrix entries (Methods 2.5). Finally, we performed a parametric bootstrap procedure on the reduced payoff matrix to determine which of the remaining interaction coefficients were significantly different from zero (Methods 2.6). This process produced a final payoff matrix of estimated frequency-dependent interaction coefficients, yielding a parsimonious representation of the dynamics.

To evaluate our framework’s performance, we generated 1,000 synthetic time-series datasets. The process began by creating a sparse random payoff matrix, $M^{*}$, with entries $M_{ij}^{*}\in [-1,1]$, which was validated to ensure it produced an ESS (See Methods Sect 2.8). Using this ground-truth matrix and the replicator equation, we then generated a synthetic time series, $\Omega^{*}(n,\epsilon )$, systematically varying both the number of SPs (n) and the level of observational noise (ϵ, see Methods Sect 2.8).

**Fig 3:**
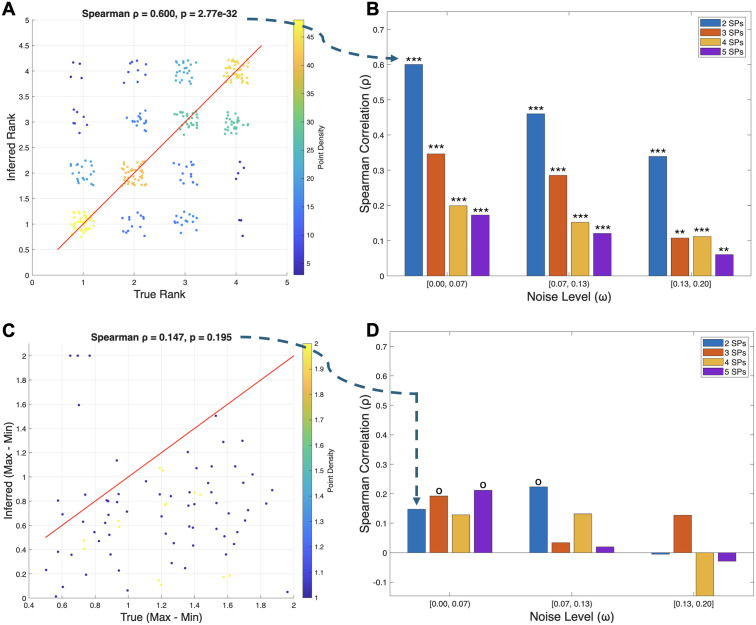
Comparative analysis of framework performance on inferring payoff matrix rank and range. **(A)** A density scatter plot correlation between true and inferred payoff matrix entry ranks for the 2-player, low-noise condition (ρ=0.600, p≪0.001). **(B)** Spearman correlations remain significant as noise and the number of SPs increase, though the effect size diminishes. **(C)** Scatter plot for the inference of payoff matrix range (max-min) for the same 2-player, low-noise condition (ρ=0.147, p=0.195). **(D)** Performance in range inference is shown across all conditions. Spearman rank correlation: o p<0.1 * p<0.05, ** p<0.01, *** p<0.001.

From each synthetic time series, we inferred a payoff matrix, M, and assessed the framework’s ability to recover two key properties of the ground-truth matrix, $M^{*}$: first, the rank order of its entries, and second, its range (the difference between the maximum and minimum values). The framework demonstrated high proficiency in recovering the rank order of interactions (Fig 3A–3B). For instance, in the low-noise, 2-SP condition, the Spearman rank correlation between true and inferred entries was strong and highly significant (ρ=0.600, p≪0.001; Fig 3A). This robust performance was consistent across all conditions; while the correlation strength decreased with added noise and dimensionality, it remained significant in all cases (Fig 3B). In sharp contrast, the framework was unable to reliably infer the range of the payoff matrix entries (Fig 3C–3D). For the same low-noise, 2-SP condition, the correlation between the true and inferred range was weak and not statistically significant (ρ=0.147,p=0.195; Fig 3C). This poor performance was systematic, with the correlation failing to reach significance in most conditions (Fig 3D). Taken together, these results indicate that while our framework can robustly determine the relative importance and hierarchy of interactions, it does not capture their absolute magnitudes from the time-series data alone. Specifically, as illustrated in Fig 3C, the inferred range of payoff values—and their associated uncertainty intervals—can underestimate the true underlying range by approximately a factor of two, indicating that ECO-K estimates should be interpreted in relative rather than absolute terms.

### 3.2 Model-based inference of frequency-dependent interactions in TNBC

Copy number alterations play a critical role in cancer development and progression, impacting cellular phenotypes and patient outcomes. Shah and colleagues [15] recently performed serial-passaging (Fig 1) experiments followed by scWGS of TNBC cell lines and PDX tumor mouse models, allowing for longitudinal copy number inference. *In-vitro* and *in-vivo* lineages were cultured for 20–60 passages and sampled four to seven times for sequencing and copy number inference. A subset of lineages were exposed to cisplatin at various passages to evaluate whether the drug selects for certain copy number alterations.

We applied ECO-K to these TNBC cell lines and PDXs to estimate interaction coefficients under a frequency-dependent selection model. ECO-K assumes that subpopulation fitness depends on the frequencies of other subpopulations, and infers a payoff matrix that best explains the observed SP frequency trajectories. We grouped 10 lineages into seven replicate groups (Table 1). Two lineages $X_{i}$ and $X_{j}$ were considered replicates (i.e., members of the same replicate group), if and only if they originated from the same PDX or cell line and had been exposed to cisplatin for the same number of passages (Sect 2.2). We clustered single-cell genomes across all replicates within a given replicate group by their karyotype to define SPs (Sect 2.2), which resulted in an average of four SPs (between two and seven) per replicate. Thus if we assumed interactions between every pairwise set of SPs, ECO-K would find 55 non-zero entries across seven payoff matrices.

Applying ECO-K to these seven replicate groups, we assigned 39 interaction coefficients in total with an average of six interaction coefficients per group (Table 1). Parametric bootstrapping confirmed that 36 (92%) of these interactions estimated coefficients were consistently non-zero under the assumed model (Table 1). We considered a replicate group to be well described by the frequency-dependent model if at least one replicate had a RMSE (i.e., difference between predicted and observed frequency trajectories) less than or equal to 0.05 and the matrix representing the inferred interactions between SPs in that replicate group had a mean absolute matrix entry (i.e., effect size) above 0.10. Out of seven replicate groups, six met these criteria. Interestingly, untreated cases had a 2.87 times higher proportion of positive matrix entries compared to cases exposed to Cisplatin (t-test: p-value = 0.0483; S4 Fig), suggesting that competitive dynamics are less intense under cisplatin exposure.

To clarify whether intrinsic growth differences alone between SPs could explain our observations, we compared the ECO-K framework introduced here to the FitClone method [15]. FitClone uses a Bayesian Wright-Fisher diffusion model, which estimates constant growth rates (referred to as selection coefficients) for each SP independently. Importantly, FitClone assumes that the growth rate of each SP is unaffected by the presence or frequency of other SPs within the tumor. In contrast, ECO-K explicitly captures frequency-dependent selection through a payoff matrix, where each entry quantifies how the presence of one SP affects the growth of another. Non-zero entries in the payoff matrix represent meaningful interactions between specific pairs of SPs, while zero entries imply interactions which do not have an effect on fitness. Here, each SP represents a distinct karyotypic configuration, analogous to a “strategy” in evolutionary game theory. Fits to all replicate groups are given in S5 Fig.

**Fig 4:**
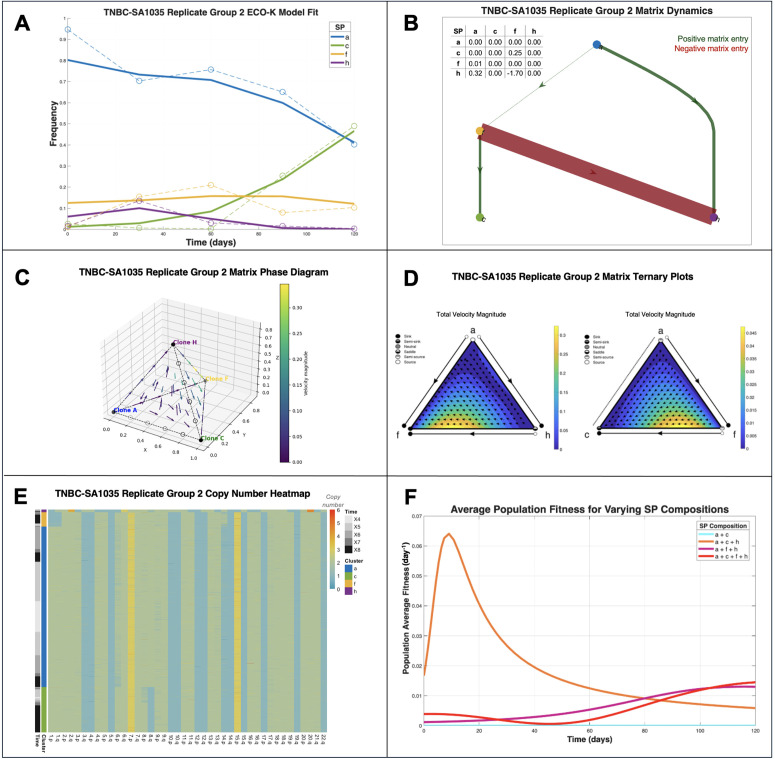
Estimated frequency-dependent interaction coefficients between karyotype defined subpopulations in TNBC PDX mouse models. **(A)** Observed (dashed lines) and replicator equation–predicted frequency dynamics (solid lines) for subpopulations (SPs) in TNBC-SA1035 Replicate Group 2. **(B)** Payoff matrix diagram illustrating interactions between SPs. Red bands represent a negative payoff matrix entry, green bands represent a positive entry. The width of the band represents the relative strength of the interaction, and the arrow gives the direction of the interaction (i.e., which SP receives the payoff). **(C)** Replicator phase diagram correlating to **(B). (D)** Ternary plots of replicator dynamics for selected SP combinations: A–F–H (left) and A–C–F (right). **(E)** Heatmap of copy number alterations (CNAs) across SPs, annotated by sampling timepoint and cluster. **(F)** Mean population fitness ($\bar{f_{t}}$ from Eq 3) trajectories under different SP compositions. Removing specific SPs alters overall fitness relative to the original composition (red).

Among six replicate groups whose dynamics were well explained by the frequency-dependent model, two could be better fit by the baseline frequency-independent FitClone model (TNBC-SA1035 Replicate Groups 1 and 2), and two were better fit by ECO-K (TNBC-SA609 Replicate Groups 5 and 6), while the remaining two (TNBC-SA535 Replicate Group 4 and 184-hTERT p53KO) had insufficient SPs (must be >2) to be evaluated by FitClone (Table 1). We defined ‘better fit’ as having both a lower AICc and a lower BIC compared to the alternative model (Table 1). RMSE values are reported descriptively but were not used as the primary model selection criterion. Here we highlight one lineage where the frequency-dependent model provided a particularly good description of the observed dynamics (Fig 4): Replicate Group 2 in PDX TNBC-SA1035 (Fig 4).

In TNBC-SA1035 Replicate Group 2, the best fit payoff matrix for the four karyotype-defined SPs (Fig 4E; Sect 2.2.2), had four non-zero entries (Fig 4B). The most notable changes occur between days 60 and 120 (Fig 4A), corresponding to the trajectory where the dominant subpopulation transitions from SP A to SP C. This is illustrated in a total velocity magnitude ternary plot (Fig 4D, Methods Sect 2.7). In Fig 4D, we focus in on the A-H-F and A-C-F trilateral relationships to demonstrate how SP F (the only SP assigned non-zero coefficients relative to all other SPs) influences the evolutionary trajectory of the population towards dominance of SP C.

Potential mechanisms for interactions arise from analyzing the replicator dynamics and copy number profiles of the subpopulations (Fig 4E). Subpopulation F was the only subpopulation that interacted with all three other SPs (Fig 4B–4D) and was characterized by chromosome 1 loss and gain of chromosome 14p (Fig 4E). Chromosome 1 harbors multiple genes involved in metabolism (e.g., *GLUL*, involved in glutamine metabolism and NADPH-dependent enzymes) [17,18]. Its loss might impair SP F’s ability to synthesize certain metabolites, making it more dependent on metabolic exchange with other SPs, facilitating broad interactions.

Alternatively, if we consider specifically SP F’s interaction with SP C: SP F’s gain of 14p (Fig 4E) may promote SP C due to upregulation of *TGFB3* [19], a signaling factor that supports proliferation and immune evasion [20,21]. This compensates for SP C’s loss of 8q, which may have impaired its autonomous survival metabolic flexibility [22].

Collectively, these results support the hypothesis that karyotypic alterations can drive frequency-dependent interactions in specific contexts (Table 1).

## 4 Discussion

In this study, we introduce a novel inverse game-theoretic framework capable of estimating frequency-dependent interaction coefficients among karyotypically distinct tumor subpopulations (SPs). This approach leverages single-cell whole-genome sequencing data to identify candidate interactions that may shape a tumor’s evolutionary trajectory. Frequency-dependent interactions provided a better fit in two of the four analyzed datasets, whereas frequency-independent baseline fitness was favored in the remaining two, suggesting that the dominant evolutionary driver varies by lineage. Our framework therefore serves as a hypothesis-generating engine, suggesting that tumor ecosystems may be organized around ecological hubs—subpopulations whose interactions are critical for community stability. For example, in the PDX TNBC-SA1035 model, our analysis predicts that subpopulation F may act as such a hub (Fig 4B–4D). This leads to a clear, testable hypothesis: targeted removal or inhibition of SP F would disproportionately disrupt the growth dynamics of the entire tumor ecosystem.

The specific genomic alterations of this candidate hub—loss of chromosome 1 and gain of 14p (Fig 4E)—point toward potential mechanisms. The loss of chromosome 1 may impair metabolic functions like glutamine biosynthesis, forcing SP F into a state of ecological dependency on other subpopulations. This leads to a second, therapeutically relevant hypothesis: targeting glutamine metabolism [23–25] would function as a selective strategy to dismantle SP F’s hub-like role and destabilize the tumor network. Cisplatin exposure was associated with a shift in the pattern of estimated coefficients, suggesting that cooperative effects may increase under treatment. If validated, this suggests that the tumor’s ecological network is not static but a druggable, dynamic entity, opening avenues to test combinatorial or sequential therapies designed to first reshape, then exploit, these dependencies.

However, our study does carry limitations which should be considered. Firstly, we acknowledge that cell phenotype and behavioral diversity can arise from both genetic (e.g., copy number variation) and non-genetic (epigenetic, transcriptional, microenvironmental) sources of heterogeneity. Secondly, the robustness of our inferences is constrained by the limited temporal resolution of available datasets. Crucially, our analyses revealed a fundamental identifiability limitation inherent to the data structure and the replicator dynamics model. As demonstrated in our synthetic benchmarks, the framework cannot recover the absolute magnitude (or range) of payoff entries. This is not merely an empirical shortcoming: the replicator equation describes changes in relative frequencies, which depend only on differences in fitness terms. Any payoff matrix can be transformed by adding a constant to each column or by multiplying the entire matrix by a positive scalar without altering the resulting trajectories (a property known as ’strategic equivalence’). Because of this, while ECO-K can robustly infer the relative ordering, sign, and hierarchical importance of interaction coefficients, it cannot assign biologically meaningful absolute strengths to those effects. This same mathematical limitation explains why attempts to separate frequency-independent baseline fitness from frequency-dependent payoffs proved unidentifiable in a hybrid fit to the data (S8 Fig). Indeed, forcing a hybrid fit to such data not only failed to recover baseline rates but also degraded the accuracy of the inferred interaction coefficients (decreasing rank correlation from 0.60 to 0.45 in synthetic benchmarks). With frequency data alone, any clone-specific baseline growth rate cancels out of the replicator dynamics, making it impossible to disentangle baseline fitness from frequency-dependent effects without additional experimental measurements (e.g., absolute cell counts or monoculture growth assays). Consistent with this theoretical constraint, our synthetic benchmarks show that ECO-K systematically underestimates the true range of payoff values—and even the parametric bootstrap intervals remain narrower than the ground-truth spread (Fig 3C–3D). This emphasizes that ECO-K should be used to interpret relative patterns (sign structure and ranking) of coefficients rather than their absolute magnitudes.

Addressing these limitations would require experiments specifically designed to validate frequency-dependent interactions driven by karyotypic alterations. Sorting individual SPs from their mixed populations and measuring their absolute growth rate over time in isolation would inform de-convolution of frequency-dependent and independent effects. Future studies could adopt targeted perturbation experiments, involving the removal of specific subpopulations to observe impacts on tumor growth rates and evolutionary trajectories. This could be achieved through the administration of cisplatin, which has differential efficacy across karyotypes [15], that would provide the opportunity to evaluate the existence of SPs acting as interaction hubs (Fig 4B–4C) and validate our predictions regarding changing tumor growth dynamics as a function of subpopulation composition (Fig 4E). Enhanced experimental resolution through higher temporal sampling and integration of multimodal data (e.g., transcriptomics, metabolomics) would also deepen our understanding of frequency-dependent dynamics and the phenotypic consequences of karyotypic alterations in tumor evolution.

Our work suggests that frequency-dependent selection between cancer subpopulations, potentially influenced by distinct karyotypic profiles, may be empirically assessed *in-vivo* and *in-situ*. The inferred fitness values could help guide therapeutic strategies aimed at exploiting or mitigating intratumor ecological dynamics by strategically targeting key subpopulations to hinder cancer progression.

## Supporting information

S1 Text Supplementary methods and supporting analyses. This document provides extended methodological detail and additional analyses supporting the main manuscript. It includes: (i) correlation analysis linking subpopulation growth rates to subpopulation frequencies; (ii) likelihood-based parameterization of the payoff matrix using a negative log-likelihood framework; (iii) simulation-based assessment of convergence to an evolutionarily stable strategy (ESS) in artificially generated datasets; (iv) evaluation of payoff-matrix inference performance in synthetic data (including recovery of entry ranks and dynamic range); (v) comparative analysis of inferred payoff matrices in treated versus untreated datasets; (vi) cross-dataset comparison of fitted trajectories, including ECO-K versus FitClone where applicable; (vii) clustering robustness diagnostics (k-means stability metrics, including silhouette-based measures); (viii) sensitivity analysis of interaction-ranking procedures; and (ix) a theoretical derivation and empirical illustration of the unidentifiability limits of a hybrid model combining intrinsic fitness with frequency-dependent payoff terms.
(PDF)

S1 Fig Distance to ESS over time for 1,000 artificially generated datasets. The horizontal axis indexes each dataset, while the vertical axis represents time from 0 to 50. Colors denote distance from the ESS (blue indicating smaller distances, yellow larger distances). Each vertical “column” thus shows how quickly and closely a particular solution approaches its ESS over the simulated time span.
(TIFF)

S2 FigPerformance of the payoff matrix inference model.Density scatter plots comparing the true rank versus the inferred rank of matrix entries, decomposed by the number of interacting SPs and the level of observational noise. Each point represents an entry from one of 1000 simulated datasets, with color indicating the local density of points. The Spearman’s rank correlation coefficient (ρ) and p-value are shown for each condition, revealing a general decrease in rank inference quality with increasing noise and dimensionality.(TIFF)

S3 FigDensity of Max-Min comparisons across varying conditions.A grid of scatter plots illustrating the relationship between the true range (maximum - minimum) of the data and the inferred range, decomposed by the number of SPs and the level of noise (Ω). Each panel shows the density of data points, with warmer colors indicating a higher concentration of simulations falling within that region. The red line in each subplot represents the line of perfect agreement (y=x). Within each subplot, the Spearman rank correlation coefficient (ρ) and its corresponding p-value (p) are displayed, quantifying the linear relationship between the true and inferred ranges under that specific condition.(TIFF)

S4 Fig Analysis of matrices in treated versus untreated datasets. The distribution of four key metrics between treated and untreated sample groups: average magnitude, average value, positive fraction, and negative fraction of payoff matrix entries. Violin plots illustrate the data distribution for each group, with individual data points shown as black dots. A black line represents the interquartile range (IQR), and a white dot with a black outline indicates the median. P-values from an independent samples t-test are included in the title of each subplot to indicate the statistical significance of the difference between the groups for each metric.
(TIFF)

S5 Fig Fits for all datasets. ECO-K fits are shown alongside corresponding FitClone fits (left panels) for replicate groups with three or more subpopulations (SPs), including TNBC-SA1035 (Replicate Groups 1–2) and TNBC-SA609 (Replicate Groups 1–2). For replicate groups with only two SPs (TNBC-SA535 Replicate Groups 1–2 and hTERT-184 p53 KO Replicate Group 1), only ECO-K fits are shown, as FitClone requires more than two SPs for inference. This comparison highlights the consistency and differences in inferred SP dynamics between the two approaches.
(TIFF)

S6 Fig SP k-means clustering analysis. **(A)** For the hTERT-184 p53 KO cell line, the total within-cluster sum of squares was identical, and the mean silhouette was 0.841 (CV = 0%). All 25 runs converged to the same optimum, indicating a uniquely stable clustering for this dataset. **(B)** For TNBC-SA535, the total WCSS varied by 4.7%, and the mean silhouette width varied by 0.058 (CV = 20%). 18 of 25 runs converged to two high-quality, closely related optima. **(C)** TNBC-SA609, the WCSS varied by only 2.3%, and the mean silhouette width varied by 0.027 (CV = 12%). 17 of 25 runs converged to two nearly identical optima. **(D)** For TNBC-SA1035, the total WCSS varied by 4.7%, and the mean silhouette width varied by 0.031 (CV = 9.8%). 22 of 25 runs converged to two nearly identical optima.
(TIFF)

S7 Fig SP interaction sensitivity analyses. Analyses of SP frequency data from *in-vitro* evolution experiments to find significant growth interactions between different SPs. A two sample t-test (MATLAB R2025a “ttest2” function) was used to calculate interaction strengths and p-values, followed by two distinct methods to select the most significant interactions: one based on a composite significance score (Methods Sect 2.3), the other on p-values alone.
(TIFF)

S8 FigHybrid model performance illustrating the identifiability limits of separating intrinsic (baseline) fitness from frequency-dependent payoff terms.(**A**) Recovery of intrinsic fitness ranks under the hybrid model for synthetic 2-SP systems across increasing noise levels. In all cases, the model fails to recover the true intrinsic fitness order (Spearman’s ρ ≈ 0; p > 0.05), demonstrating that baseline fitness cannot be reliably inferred from relative-frequency trajectories alone. (**B**) Recovery of payoff matrix entry ranks when both intrinsic fitness and frequency-dependent effects are present. Although payoff inference remains partially informative (ρ 0.43–0.45), performance is degraded compared to the frequency-dependent-only setting (Fig 2B in main text). Across noise regimes, the hybrid model systematically fails to disentangle intrinsic fitness from payoff contributions, resulting in lower rank accuracy.(TIFF)

## References

[1] Nowell PC. The clonal evolution of tumor cell populations. Science. 1976;194(4260):23–8. doi: 10.1126/science.959840 959840

[2] Basanta D, Anderson ARA. Exploiting ecological principles to better understand cancer progression and treatment. Interface Focus. 2013;3(4):20130020. doi: 10.1098/rsfs.2013.0020 24511383 PMC3915838

[3] Bakhoum SF, Ngo B, Laughney AM, Cavallo J-A, Murphy CJ, Ly P, et al. Chromosomal instability drives metastasis through a cytosolic DNA response. Nature. 2018;553(7689):467–72. doi: 10.1038/nature25432 29342134 PMC5785464

[4] Sansregret L, Vanhaesebroeck B, Swanton C. Determinants and clinical implications of chromosomal instability in cancer. Nat Rev Clin Oncol. 2018;15(3):139–50. doi: 10.1038/nrclinonc.2017.198 29297505

[5] Zack TI, Schumacher SE, Carter SL, Cherniack AD, Saksena G, Tabak B, et al. Pan-cancer patterns of somatic copy number alteration. Nat Genet. 2013;45(10):1134–40. doi: 10.1038/ng.2760 24071852 PMC3966983

[6] Shukla A, Nguyen THM, Moka SB, Ellis JJ, Grady JP, Oey H, et al. Chromosome arm aneuploidies shape tumour evolution and drug response. Nat Commun. 2020;11(1):449. doi: 10.1038/s41467-020-14286-0 31974379 PMC6978319

[7] Ferrall-Fairbanks MKG. Modeling adaptive therapy in non-muscle invasive bladder cancer. bioRxiv. 2019. https://www.biorxiv.org/content/10.1101/826438v2

[8] Folkman J. The role of angiogenesis in tumor growth. Semin Cancer Biol. 1992;3(2):65–71. 1378311

[9] Mansury Y, Deisboeck TS. The impact of “search precision” in an agent-based tumor model. J Theor Biol. 2003;224(3):325–37. doi: 10.1016/s0022-5193(03)00169-3 12941591

[10] Basanta D, Deutsch A. A game theoretical perspective on the somatic evolution of cancer. Modeling and Simulation in Science, Engineering and Technology. Birkhäuser Boston. 2008. p. 1–16. 10.1007/978-0-8176-4713-1_5

[11] Mansury Y, Diggory M, Deisboeck TS. Evolutionary game theory in an agent-based brain tumor model: exploring the “Genotype-Phenotype” link. J Theor Biol. 2006;238(1):146–56. doi: 10.1016/j.jtbi.2005.05.027 16081108

[12] Tomlinson IP, Bodmer WF. Modelling the consequences of interactions between tumour cells. Br J Cancer. 1997;75(2):157–60. doi: 10.1038/bjc.1997.26 9010019 PMC2063276

[13] Kaznatcheev A, Peacock J, Basanta D, Marusyk A, Scott JG. Fibroblasts and alectinib switch the evolutionary games played by non-small cell lung cancer. Nat Ecol Evol. 2019;3(3):450–6. doi: 10.1038/s41559-018-0768-z 30778184 PMC6467526

[14] Freischel AR, Damaghi M, Cunningham JJ, Ibrahim-Hashim A, Gillies RJ, Gatenby RA, et al. Frequency-dependent interactions determine outcome of competition between two breast cancer cell lines. Sci Rep. 2021;11(1):4908. doi: 10.1038/s41598-021-84406-3 33649456 PMC7921689

[15] Salehi S, Kabeer F, Ceglia N, Andronescu M, Williams MJ, Campbell KR, et al. Clonal fitness inferred from time-series modelling of single-cell cancer genomes. Nature. 2021;595(7868):585–90. doi: 10.1038/s41586-021-03648-3 34163070 PMC8396073

[16] West J, Ma Y, Kaznatcheev A, Anderson ARA. IsoMaTrix: a framework to visualize the isoclines of matrix games and quantify uncertainty in structured populations. Bioinformatics. 2021;36(22–23):5542–4. doi: 10.1093/bioinformatics/btaa1025 33325501 PMC8016459

[17] Sandhu MS, Waterworth DM, Debenham SL, Wheeler E, Papadakis K, Zhao JH, et al. LDL-cholesterol concentrations: a genome-wide association study. Lancet. 2008;371(9611):483–91. doi: 10.1016/S0140-6736(08)60208-1 18262040 PMC2292820

[18] Wang Y, Kudoh J, Kubota R, Asakawa S, Minoshima S, Shimizu N. Chromosomal mapping of a family of human glutamine synthetase genes: functional gene (GLUL) on 1q25, pseudogene (GLULP) on 9p13, and three related genes (GLULL1, GLULL2, GLULL3) on 5q33, 11p15, and 11q24. Genomics. 1996;37(2):195–9. doi: 10.1006/geno.1996.0542 8921392

[19] Shiying S, Weihong W, Xiuqiong T, Yemei Q. TGFB3 gene mutation associated with mandibular coronoid process hyperplasia: a family investigation. Oral Surg Oral Med Oral Pathol Oral Radiol. 2023;136(2):e109–15. doi: 10.1016/j.oooo.2023.04.004 37246056

[20] Tauriello DVF, Sancho E, Batlle E. Overcoming TGFβ-mediated immune evasion in cancer. Nat Rev Cancer. 2022;22(1):25–44. doi: 10.1038/s41568-021-00413-6 34671117

[21] Jonson T, Albrechtsson E, Axelson J, Heidenblad M, Gorunova L, Johansson B, et al. Altered expression of TGFB receptors and mitogenic effects of TGFB in pancreatic carcinomas. Int J Oncol. 2001. doi: 10.3892/ijo.19.1.71 11408925

[22] Vízkeleti L, Spisák S. Rewired metabolism caused by the oncogenic deregulation of MYC as an attractive therapeutic target in cancers. Cells. 2023;12(13):1745. doi: 10.3390/cells12131745 37443779 PMC10341379

[23] Zou W, Han Z, Wang Z, Liu Q. Targeting glutamine metabolism as a potential target for cancer treatment. J Exp Clin Cancer Res. 2025;44(1):180. doi: 10.1186/s13046-025-03430-7 40598593 PMC12210561

[24] Hao Y. Computation and analysis of evolutionary game dynamics. Ames, IA: Iowa State; 2013. https://dr.lib.iastate.edu/server/api/core/bitstreams/2bff108e-32a6-47e7-afda-a46a11fddf6f/content

[25] Zar JH. Biostatistical analysis. Always learning. Pearson Education Limited; 2014. https://books.google.com/books?id=OhYCngEACAAJ

