# Supplementary material for: Inverse game theory characterizes frequency-dependent selection driven by karyotypic diversity in triple-negative breast cancer
Source: PLoS Comput Biol. 2026 Mar 10;22(3):e1013897. doi: 10.1371/journal.pcbi.1013897 (PMC13108871; doi:10.1371/journal.pcbi.1013897)
Supplement: S1 Text — This document provides extended methodological detail and additional analyses supporting the main manuscript. It includes: (i) correlation analysis linking subpopulation growth rates to subpopulation frequencies; (ii) likelihood-based parameterization of the payoff matrix using a negative log-likelihood framework; (iii) simulation-based assessment of convergence to an evolutionarily stable strategy (ESS) in artificially generated datasets; (iv) evaluation of payoff-matrix inference performance in synthetic data (including recovery of entry ranks and dynamic range); (v) comparative analysis of inferred payoff matrices in treated versus untreated datasets; (vi) cross-dataset comparison of fitted trajectories, including ECO-K versus FitClone where applicable; (vii) clustering robustness diagnostics (k-means stability metrics, including silhouette-based measures); (viii) sensitivity analysis of interaction-ranking procedures; and (ix) a theoretical derivation and empirical illustration of the unidentifiability limits of a hybrid model combining intrinsic fitness with frequency-dependent payoff terms. (PDF) [file pcbi.1013897.s001.pdf]

5 Supporting information

5.1 Correlation between growth rate and subpopulation frequency

Recall that $T = \{t_1, t_2, ...t_\tau\}$ is defined as all timepoints (i.e., passages) across replicates for a given replicate group $\Omega := \{\mathbf{X_{r1}}, \mathbf{X_{r2}}, \dots\}$.Then, $\forall t \in T$, we defined the growth rate α_{it} of SP i between passages t and $t+1$ by the difference in the natural logarithm of its frequencies, normalized by the difference in days:

$$\alpha_{it} \;=\; \frac{\log\bigl(x_{i,t+1}\bigr) \;-\; \log\bigl(x_{it}\bigr)}{dt} \quad \text{for} \quad t \;=\; 1, 2, \dots, \tau - 1. \tag{8}$$

where dt is the difference between passage t and the subsequent passage in units of days. For each pair of subpopulations (q, v), we sought to determine whether the frequency of SP v influenced the growth rate of SP q. We therefore computed the Pearson correlation coefficient R_{qv} by correlating:

$$\bigl\{\,\alpha_{q,1}, \alpha_{q,2}, \dots, \alpha_{q,\tau-1}\bigr\} \quad \text{with} \quad \bigl\{\,x_{v,1}, x_{v,2}, \dots, x_{v,\tau-1}\bigr\}.$$

Hence,

$$R_{qv} \;=\; \mathrm{corr}\Bigl(\{\alpha_{qt}\}_{t=1}^{\tau-1}, \{\,x_{vt}\}_{t=1}^{\tau-1}\Bigr). \tag{9}$$

We used a t-test to obtain a corresponding p-value P_{qv} for the null hypothesis of zero correlation.

We then applied a Bonferroni correction across all $n \times n$ pairwise tests, i.e.,

$$\widetilde{P}_{qv} \;=\; \min\Bigl(P_{qv} \times (n \cdot n),\; 1\Bigr).$$

We defined a significance score $\mathcal{S}_{qv}$ by combining the (Bonferroni-corrected) p-value $\widetilde{P}_{qv}$ and the magnitude of the correlation:

$$\mathcal{S}_{qv} \;=\; -\log_{10}\bigl(\widetilde{P}_{qv}\bigr) \;\times\; \bigl|\,\overline{R}_{qv}\bigr|. \tag{10}$$

Higher scores indicate stronger frequency-dependent interaction between SPs q and v. Ranking interactions by p-value alone did not change the interaction ranks (S7 Fig).

Following [24], we impose an upper limit on the number of potentially non-zero payoff matrix entries. Specifically, if there were n SPs in the dataset, at most $(2n-1)$ such pairwise interactions were retained as candidates for being non-zero in the payoff matrix. This restriction is motivated by the need to avoid overfitting and ensure identifiability of payoff matrix entries. To implement this threshold, we rank the (q, v) pairs in descending order of $\mathcal{S}_{qv}$ and select the top $(2n-1)$. All other entries in the payoff matrix were then set to zero. The sign of the correlation $(\pm)$ was used to determine whether the selected payoff entry should be positive or negative, ensuring that both the magnitude and direction of the interaction were captured.

5.2 Parametrization based on negative log-likelihood

The probability of observing replicate $\mathbf{X} \in \Omega$ given the payoff matrix M can be modeled by a normal distribution of residuals:

$$P(\mathbf{X} \mid M) = \prod_{j=1}^{n} \prod_{t=1}^{\tau} \mathcal{N}(d_{jt} \mid 0, \sigma_j^2), \tag{11}$$

where $d_{jt} = \mathbf{X_{jt}} - x_{jt}$ represents the residuals, with $\mathbf{X_{jt}}$ being the observed frequency of SP j at timepoint t, and x_{jt} being the predicted frequency based on solution to Eqn. 3 given the payoff matrix M and initial conditions $x(0) = \frac{1}{3}\sum_{t=1}^{3} \mathbf{X}_{:,t}$. The initial frequency for each SP was calculated as the average of its observed frequencies across the first three timepoints in the dataset. This averaging helped to reduce noise or measurement error that might be present at a single timepoint and provided a more stable starting point for the simulation.

The variance σ_j^2 for each SP j was estimated as $\sqrt{\frac{1}{4G}}$ [25], where G is the effective number of cell representatives of SP j . While the average number of cells sequenced per timepoint in our dataset was 1146 [15], we set $G = 100$ to reflect the scale of the smallest observed SP (approximately 10% of the total population).

The overall log-likelihood for replicate $\mathbf{X}$ is then:

$$\ln P(\mathbf{X} \mid M) = -\frac{1}{2} \sum_{j=1}^{n} \sum_{t=1}^{\tau} \left[\ln(2\pi\sigma_j^2) + \frac{(d_{jt})^2}{\sigma_j^2} \right]. \tag{12}$$

The cumulative negative log-likelihood across all replicates was calculated as:

$$\mathcal{L} = -\sum_{X \in \Omega} \ln P(\mathbf{X} \mid M). \tag{13}$$

5.3 ESS convergence across artificially generated datasets

To assess the velocity of our artificial data towards the ESS, we recorded the distance from the ESS state over time. We generated 1,000 random payoff matrices and solved the replicator equations over time as previously described in Section 2.8. We then verified that the final state was indeed at or near an Evolutionarily Stable Strategy (ESS) by checking the relevant stability conditions. For each solution, we measured the distance of the evolving population state from the ESS at each timepoint and store these distances. Finally, we compiled all 1,000 distance-to-ESS trajectories into a single figure to visualize how quickly or slowly each population converges toward its ESS.

To ensure that the terminal state was at or near an ESS, we required:

- That the difference between the maximum and minimum fitness values (rounded to one decimal place) at the end of the simulation be at most 0.1:

$$\max f_i(\mathbf{x}) - \min f_i(\mathbf{x}) \leq 0.10, \tag{14}$$

- And that all non-trivial eigenvalues of the Jacobian matrix,

$$J_{ij} = \frac{\partial}{\partial x_j} \left[x_i \big(f_i(\mathbf{x}) - \bar{f}(\mathbf{x}) \big) \right], \tag{15}$$

  had negative real parts (as checked numerically).

If these criteria were satisfied, we regarded the final state of the replicator dynamics as nearing convergence to an ESS. While each system converged near an ESS, the speed at which it converged varied across artificial datasets (S1 Fig).

From the simulation results, we focused on timepoints up to $t = 30$ and sampled 10 equally spaced points over this interval. At each of these timepoints, we recorded the SP frequencies $\mathbf{x_{jt}} \in \mathbf{X}$. We then introduced observational noise ω drawn uniformly from $[0, 0.2]$ (i.e., $\omega \in [0, 0.2]$), and perturbed each entry $\mathbf{x_{jt}}$ with an i.i.d. Gaussian random variable $\eta_{jt} \sim \mathcal{N}(0, \omega)$. Specifically, the "noisy" version $\mathbf{X'}$ was computed element-wise as:

$$\mathbf{x'_{jt}} = \frac{\max\big(0,\ \mathbf{x_{jt}} + \eta_{jt}\big)}{\sum_k \max\big(0,\ \mathbf{x_{kt}} + \eta_{kt}\big)}. \tag{16}$$

Thus, any negative values after noise addition were set to zero, and each timepoint was subsequently renormalized so that SP fractions sum to one. Finally, we applied our payoff recovery procedure to infer a payoff matrix M' from the noisy dataset $\mathbf{X}'$.

5.4 Inference of payoff matrix entry range and ranks via ECO-K in the artificially generated datasets

To evaluate our framework's performance, we generated 1,000 synthetic time-series datasets (see Methods Section 2.8). Using this ground-truth matrix and the replicator equation, we then generated a synthetic time series, $\Omega^*(n, \epsilon)$, systematically varying both the number of SPs (n) and the level of observational noise. To assess how accurately we could infer the original payoff matrix, we used the following two performance metrics: **Rank Correlation** (S2 Fig) and **Dynamic Range** (S3 Fig).

5.5 Analysis of matrices in treated versus untreated datasets

We examined the differences across the estimated coefficients between SPs in the treated and untreated settings. For every matrix fit to the experimental data, four key metrics were calculated: the mean of the absolute values of its elements (average magnitude), the standard mean (average value), the proportion of positive elements (positive fraction), and the proportion of negative elements (negative fraction). These results were then grouped by which matrices were fit to datasets which were treated with cisplatin versus those which received no treatment. The averages of these groups are reported in S4 Fig. To determine if significant differences existed between the two groups, an independent two-sample t-test (”ttest2” function in MatLab R2025a) was performed for each of the four metrics. The corresponding p-value from the t-test was included in the title of each plot to indicate the level of statistical significance:

5.6 Comparison of all fitted datasets

To evaluate the robustness of our approach, we compared ECO-K fits with those obtained from FitClone across replicate groups with three or more subpopulations (SPs). ECO-K and FitClone generally produced consistent estimates of clonal trajectories, with both methods capturing the dominant expansion and decline patterns of individual SPs. Notably, ECO-K tended to produce smoother trajectories, reflecting its explicit modeling of ecological interactions, whereas FitClone occasionally inferred more abrupt frequency shifts, particularly in cases with sparse or noisy longitudinal sampling. These differences underscore the complementary strengths of the two methods: FitClone provides a likelihood-based inference framework grounded in population genetics, while ECO-K emphasizes frequency-dependent dynamics to capture ecological competition. For replicate groups with only two SPs (TNBC-SA535 and hTERT-184 p53 KO), FitClone could not be applied, as the method requires more than two SPs to estimate relative fitness parameters.

5.7 Silhouette analysis

We selected k-means for clustering cells by their karyotype for its interpretability and suitability for discrete subclone identification in copy number space. Alternative methods, such as non-negative matrix factorization (NMF) (e.g., Gavish et al. 2023), offer a different framework for subclonal deconvolution. NMF decomposes the observed copy number matrix into a set of basis vectors (interpreted as subclonal genotypes) and corresponding weights (cellular contributions), thereby capturing both discrete and continuous clonal structures. We acknowledge that more complex methods such as NMF can provide complementary insights, especially in cases with high subclonal admixture or ambiguous cluster boundaries.

Silhouette analysis was used to evaluate the consistency and robustness of k-means clustering across replicate runs for each dataset. For each PDX or cell line, clustering was performed with 25 random starts, and both the total within-cluster sum of squares (WCSS) and the mean silhouette width were recorded to assess stability. The mean silhouette coefficient of variation (CV) was computed across runs to quantify variability in clustering quality, and the Adjusted Rand Index (ARI) was used to compare the similarity of solutions across independent initializations.

As shown in S6 Fig, clustering outcomes varied substantially between datasets. The hTERT-184 p53 KO cell line exhibited the highest stability, with all 25/25 runs converging to the same optimum (ARI = 1) with an identical WCSS and mean silhouette across runs, indicating well-separated SPs. In contrast, the PDX datasets showed greater heterogeneity in clustering solutions, consistent with more complex

underlying structure. For these datasets, most runs (17-22 out of 25) converged to one of two closely related optima (ARI > 0.90), with low discrepancy in the WCSS (varied by $< 5\%$) and mean silhouette CV ranging from $9.8 - 20\%$. Together, these results demonstrate that while the k-means approach identifies reproducible subpopulation structure in all datasets, the degree of clustering stability reflects biological and technical variability between systems.

5.8 Interaction sensitivity analysis

We performed a sensitivity analysis by comparing the ranking from our original methodology for inferring which SPs in a given sample likely interacted (Methods Section 2.3) against using p-values alone. Our goal was to determine if the top-ranked interactions were robust to this change in methodology. The results of this sensitivity analysis show a 1:1 overlap between the two ranking methods. These interactions are inferred from the time-series data before applying our iterative BIC methodology (Methods Section 2.4–2.5.

5.9 Theoretical unidentifiability of the hybrid model

The "hybrid" model proposed—combining frequency-independent intrinsic growth (r_i) and frequency-dependent interactions (A_{ij})—is mathematically unidentifiable using relative frequency data. This is due to the redundancy of parameters when the population states sum to unity ($\sum x_j = 1$). Consider the hybrid fitness function for subpopulation i:

$$f_i(x) = r_i + \sum_{j=1}^{N} A_{ij} x_j \tag{17}$$

Since , we can rewrite the intrinsic rate as an interaction term:

$$r_i = r_i \cdot 1 = r_i \sum_{j=1}^{N} x_j = \sum_{j=1}^{N} r_i x_j \tag{18}$$

Substituting this back into the fitness function:

$$f_i(x) = \sum_{j=1}^{N} r_i x_j + \sum_{j=1}^{N} A_{ij} x_j = \sum_{j=1}^{N} (A_{ij} + r_i) x_j \tag{19}$$

If we define a new matrix , the dynamics are governed entirely by:

$$f_i(x) = \sum_{j=1}^{N} A'_{ij} x_j \tag{20}$$

This demonstrates that any intrinsic growth rate r_i can be absorbed into the interaction matrix A (specifically, by adding r_i to every element of the i-th row). Consequently, there are infinite combinations of r and A that produce identical evolutionary trajectories. Attempting to fit both simultaneously (the hybrid model) represents an over-parameterized problem where the algorithm struggles to partition the signal arbitrarily between r and A, leading to the degradation of inference accuracy shown in S8 Fig.

5.9.1 Empirical limits of separating intrinsic (baseline) fitness from frequency-dependent payoff terms

References

1. Nowell PC. The clonal evolution of tumor cell populations. Science (New York, NY). 1976;194(4260):23–28.

2. Basanta D, Anderson ARA. Exploiting ecological principles to better understand cancer progression and treatment. Interface Focus. 2013;3(4):20130020. doi:10.1098/rsfs.2013.0020.

3. Bakhoum SF, Ngo B, Laughney AM, Cavallo JA, Murphy CJ, Ly P, et al. Chromosomal instability drives metastasis through a cytosolic DNA response. Nature. 2018;553(7689):467–472. doi:10.1038/nature25432.

4. Sansregret L, Vanhaesebroeck B, Swanton C. Determinants and clinical implications of chromosomal instability in cancer. Nature Reviews Clinical Oncology. 2018;15(3):139–150. doi:10.1038/nrclinonc.2017.198.

5. Zack TI, Schumacher SE, Carter SL, Cherniack AD, Saksena G, Tabak B, et al. Pan-cancer patterns of somatic copy number alteration. Nature Genetics. 2013;45(10):1134–1140. doi:10.1038/ng.2760.

6. Shukla A, Nguyen THM, Moka SB, Ellis JJ, Grady JP, Oey H, et al. Chromosome arm aneuploidies shape tumour evolution and drug response. Nature Communications. 2020;11(1):449. doi:10.1038/s41467-020-14286-0.

7. Ferrall-Fairbanks Meghan KG. Modeling adaptive therapy in non-muscle invasive bladder cancer. bioRxiv; 2019. Available from: https://www.biorxiv.org/content/10.1101/826438v2.

8. Folkman J. The role of angiogenesis in tumor growth. Seminars in Cancer Biology. 1992;3(2):65–71.

9. Mansury Y, Deisboeck TS. The impact of "search precision" in an agent-based tumor model. Journal of Theoretical Biology. 2003;224(3):325–337. doi:10.1016/s0022-5193(03)00169-3.

10. Basanta David DA. A Game Theoretical Perspective on the Somatic Evolution of cancer. Springer; 2008. Available from: https://link.springer.com/chapter/10.1007/978-0-8176-4713-1_5.

11. Mansury Y, Diggory M, Deisboeck TS. Evolutionary game theory in an agent-based brain tumor model: exploring the 'Genotype-Phenotype' link. Journal of Theoretical Biology. 2006;238(1):146–156. doi:10.1016/j.jtbi.2005.05.027.

12. Tomlinson IP, Bodmer WF. Modelling the consequences of interactions between tumour cells. British Journal of Cancer. 1997;75(2):157–160.

13. Kaznatcheev A, Peacock J, Basanta D, Marusyk A, Scott JG. Fibroblasts and alectinib switch the evolutionary games played by non-small cell lung cancer. Nature Ecology & Evolution. 2019;3(3):450–456. doi:10.1038/s41559-018-0768-z.

14. Freischel AR, Damaghi M, Cunningham JJ, Ibrahim-Hashim A, Gillies RJ, Gatenby RA, et al. Frequency-dependent interactions determine outcome of competition between two breast cancer cell lines. Scientific Reports. 2021;11(1):4908. doi:10.1038/s41598-021-84406-3.

15. Salehi S, Kabeer F, Ceglia N, Andronescu M, Williams MJ, Campbell KR, et al. Clonal fitness inferred from time-series modelling of single-cell cancer genomes. Nature. 2021;595(7868):585–590. doi:10.1038/s41586-021-03648-3.

16. West J, Ma Y, Kaznatcheev A, Anderson ARA. IsoMaTrix: a framework to visualize the isoclines of matrix games and quantify uncertainty in structured populations. Bioinformatics (Oxford, England). 2021;36(22-23):5542–5544. doi:10.1093/bioinformatics/btaa1025.

17. Sandhu MS, Waterworth DM, Debenham SL, Wheeler E, Papadakis K, Zhao JH, et al. LDL-cholesterol concentrations: a genome-wide association study. Lancet. 2008;371(9611):483. doi:10.1016/S0140-6736(08)60208-1.

18. Wang Y, Kudoh J, Kubota R, Asakawa S, Minoshima S, Shimizu N. Chromosomal Mapping of a Family of Human Glutamine Synthetase Genes: Functional Gene (GLUL) on 1q25, Pseudogene (GLULP) on 9p13, and Three Related Genes (GLULL1, GLULL2, GLULL3) on 5q33, 11p15, and 11q24. Genomics. 1996;37(2):195–199. doi:10.1006/geno.1996.0542.

19. Shiying S, Weihong W, Xiuqiong T, Yemei Q. TGFB3 gene mutation associated with mandibular coronoid process hyperplasia: a family investigation. Oral Surgery, Oral Medicine, Oral Pathology and Oral Radiology. 2023;136(2):e109–e115. doi:10.1016/j.oooo.2023.04.004.

20. Tauriello DVF, Sancho E, Batlle E. Overcoming TGFB-mediated immune evasion in cancer. Nature Reviews Cancer. 2022;22(1):25–44. doi:10.1038/s41568-021-00413-6.

21. Jonson T, Albrechtsson E, Axelson J, Heidenblad M, Gorunova L, Johansson B, et al. Altered expression of TGFB receptors and mitogenic effects of TGFB in pancreatic carcinomas. International Journal of Oncology. 2001;19(1):71–81. doi:10.3892/ijo.19.1.71.

22. Vízkeleti L, Spisák S. Rewired Metabolism Caused by the Oncogenic Deregulation of MYC as an Attractive Therapeutic Target in Cancers. Cells. 2023;12(13):1745. doi:10.3390/cells12131745.

23. Zou W, Han Z, Wang Z, Liu Q. Targeting glutamine metabolism as a potential target for cancer treatment. Journal of Experimental & Clinical Cancer Research. 2025;44(1):180. doi:10.1186/s13046-025-03430-7.

24. Hao Y. Computation and analysis of evolutionary game dynamics. Iowa State. Ames, IA; 2013. Available from: https://dr.lib.iastate.edu/server/api/core/bitstreams/2bff108e-32a6-47e7-afda-a46a11fddf6f/content.

25. Zar JH. Biostatistical Analysis. Always learning. Pearson Education Limited; 2014. Available from: https://books.google.com/books?id=OhYCngEACAAJ.

9 Supplementary Info Legend

Supporting Information S1. Supplementary Methods and Supporting Analyses. This document provides extended methodological detail and additional analyses supporting the main manuscript. It includes: (i) correlation analysis linking subpopulation growth rates to subpopulation frequencies; (ii) likelihood-based parameterization of the payoff matrix using a negative log-likelihood framework; (iii) simulation-based assessment of convergence to an evolutionarily stable strategy (ESS) in artificially generated datasets; (iv) evaluation of payoff-matrix inference performance in synthetic data (including recovery of entry ranks and dynamic range); (v) comparative analysis of inferred payoff matrices in treated versus untreated datasets; (vi) cross-dataset comparison of fitted trajectories, including ECO-K versus FitClone where applicable; (vii) clustering robustness diagnostics (k-means stability metrics, including silhouette-based measures); (viii) sensitivity analysis of interaction-ranking procedures; and (ix) a theoretical derivation and empirical illustration of the unidentifiability limits of a hybrid model combining intrinsic fitness with frequency-dependent payoff terms.

10 List of legends

S1 Fig Distance to ESS over time for 1,000 artificially generated datasets. The horizontal axis indexes each dataset, while the vertical axis represents time from 0 to 50. Colors denote distance from the ESS (blue indicating smaller distances, yellow larger distances). Each vertical "column" thus shows how quickly and closely a particular solution approaches its ESS over the simulated time span.

S2 Fig Performance of the payoff matrix inference model. Density scatter plots comparing the true rank versus the inferred rank of matrix entries, decomposed by the number of interacting SPs and the level of observational noise. Each point represents an entry from one of 1000 simulated datasets, with color indicating the local density of points. The Spearman's rank correlation coefficient (ρ) and p-value are shown for each condition, revealing a general decrease in rank inference quality with increasing noise and dimensionality.

S3 Fig Density of Max-Min Comparisons Across Varying Conditions. A grid of scatter plots illustrating the relationship between the true range (maximum - minimum) of the data and the inferred range, decomposed by the number of SPs and the level of noise (Ω). Each panel shows the density of data points, with warmer colors indicating a higher concentration of simulations falling within that region. The red line in each subplot represents the line of perfect agreement ($y = x$). Within each subplot, the Spearman rank correlation coefficient (ρ) and its corresponding p-value (p) are displayed, quantifying the linear relationship between the true and inferred ranges under that specific condition.

S4 Fig Analysis of matrices in treated versus untreated datasets. The distribution of four key metrics between treated and untreated sample groups: average magnitude, average value, positive fraction, and negative fraction of payoff matrix entries. Violin plots illustrate the data distribution for each group, with individual data points shown as black dots. A black line represents the interquartile range (IQR), and a white dot with a black outline indicates the median. P-values from an independent samples t-test are included in the title of each subplot to indicate the statistical significance of the difference between the groups for each metric.

S5 Fig Fits for all datasets. ECO-K fits are shown alongside corresponding FitClone fits (left panels) for replicate groups with three or more subpopulations (SPs), including TNBC-SA1035 (Replicate Groups 1–2) and TNBC-SA609 (Replicate Groups 1–2). For replicate groups with only two SPs (TNBC-SA535 Replicate Groups 1–2 and hTERT-184 p53 KO Replicate Group 1), only ECO-K fits are shown, as FitClone requires more than two SPs for inference. This comparison highlights the consistency and differences in inferred SP dynamics between the two approaches.

S6 Fig SP k-means clustering analysis.(A) For the hTERT-184 p53 KO cell line, the total within-cluster sum of squares was identical, and the mean silhouette was 0.841 (CV = 0%). All 25/25 runs converged to the same optimum, indicating a uniquely stable clustering for this dataset. **(B)** For TNBC-SA535, the total WCSS varied by 4.7%, and the mean silhouette width varied by 0.058 (CV = 20%). 18/25 runs converged to two high-quality, closely related optima. **(C)** TNBC-SA609, the WCSS varied by only 2.3%, and the mean silhouette width varied by 0.027 (CV = 12%). 17/25 runs converged to two nearly identical optima. **(D)** For TNBC-SA1035, the total WCSS

varied by 4.7%, and the mean silhouette width varied by 0.031 (CV = 9.8%). 22/25 runs converged to two nearly identical optima.

S7 Fig SP interaction sensitivity analyses. Analyses of SP frequency data from *in-vitro* evolution experiments to find significant growth interactions between different SPs. A two sample T-test (MatLab R2025a "ttest2" function) was used to calculate interaction strengths and p-values, followed by two distinct methods to select the most significant interactions: one based on a composite significance score (Methods Section 2.3), the other on p-values alone.

S8 Fig Hybrid model performance illustrating the identifiability limits of separating intrinsic (baseline) fitness from frequency-dependent payoff terms. (A) Recovery of intrinsic fitness ranks under the hybrid model for synthetic 2-SP systems across increasing noise levels. In all cases, the model fails to recover the true intrinsic fitness order (Spearman's $\rho \approx 0$; p $>$ 0.05), demonstrating that baseline fitness cannot be reliably inferred from relative-frequency trajectories alone. (B) Recovery of payoff matrix entry ranks when both intrinsic fitness and frequency-dependent effects are present. Although payoff inference remains partially informative (ρ 0.43–0.45), performance is degraded compared to the frequency-dependent-only setting (Fig 3B in main text). Across noise regimes, the hybrid model systematically fails to disentangle intrinsic fitness from payoff contributions, resulting in lower rank accuracy.
