# Supplementary figures and images for: Inverse game theory characterizes frequency-dependent selection driven by karyotypic diversity in triple-negative breast cancer
Source: PLoS Comput Biol. 2026 Mar 10;22(3):e1013897. doi: 10.1371/journal.pcbi.1013897 (PMC13108871; doi:10.1371/journal.pcbi.1013897)

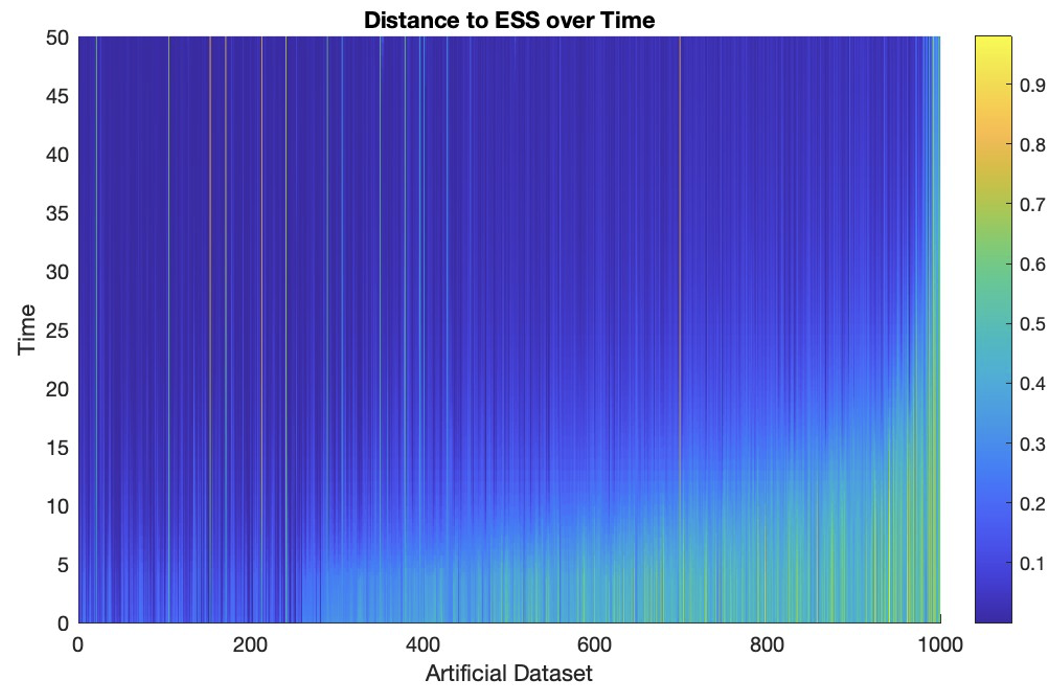

Supplement: S1 Fig — The horizontal axis indexes each dataset, while the vertical axis represents time from 0 to 50. Colors denote distance from the ESS (blue indicating smaller distances, yellow larger distances). Each vertical “column” thus shows how quickly and closely a particular solution approaches its ESS over the simulated time span. (TIFF) [file pcbi.1013897.s002.tiff]

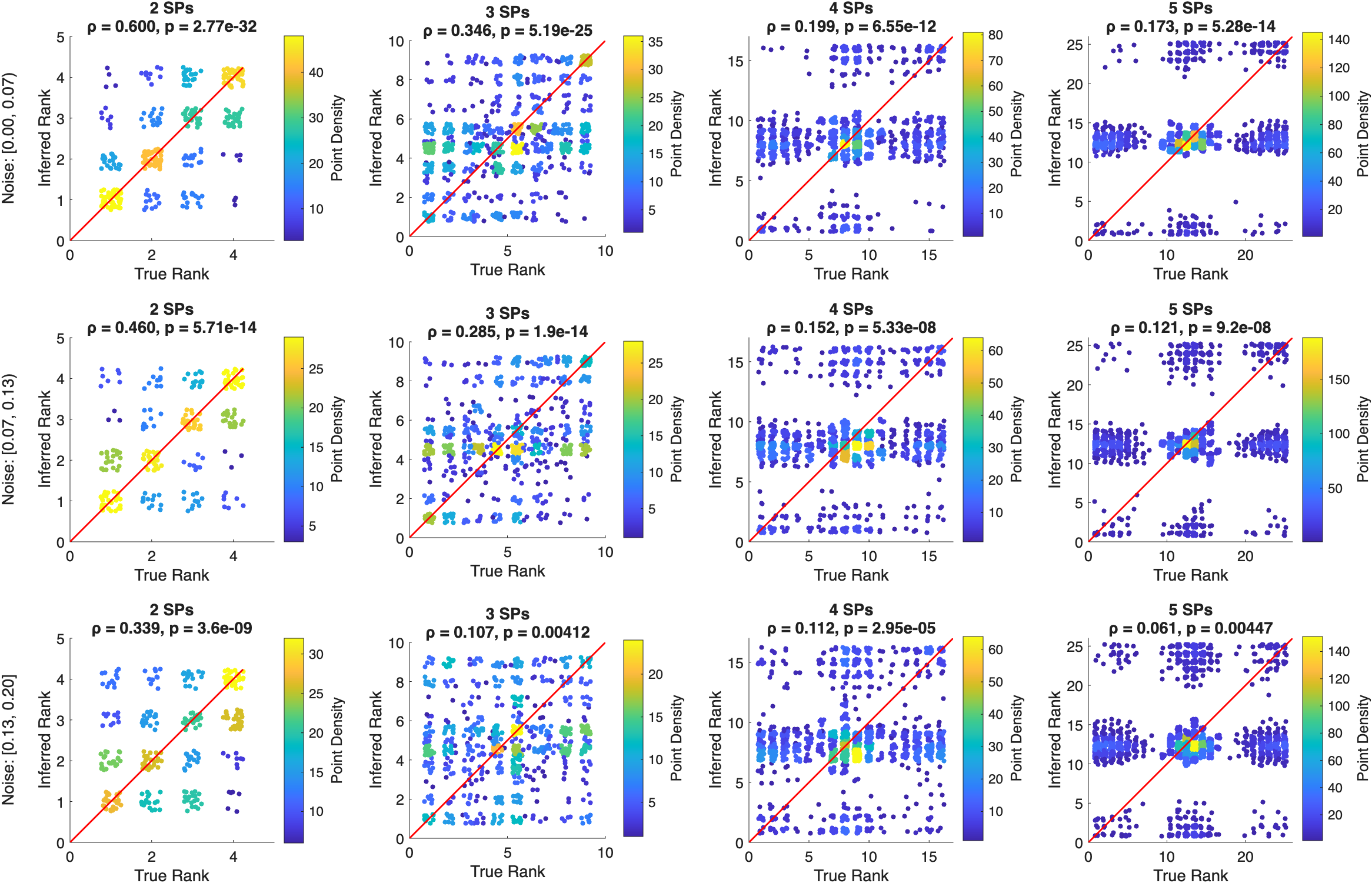

Supplement: S2 Fig — Density scatter plots comparing the true rank versus the inferred rank of matrix entries, decomposed by the number of interacting SPs and the level of observational noise. Each point represents an entry from one of 1000 simulated datasets, with color indicating the local density of points. The Spearman’s rank correlation coefficient (ρ) and p-value are shown for each condition, revealing a general decrease in rank inference quality with increasing noise and dimensionality. (TIFF) [file pcbi.1013897.s003.tiff]

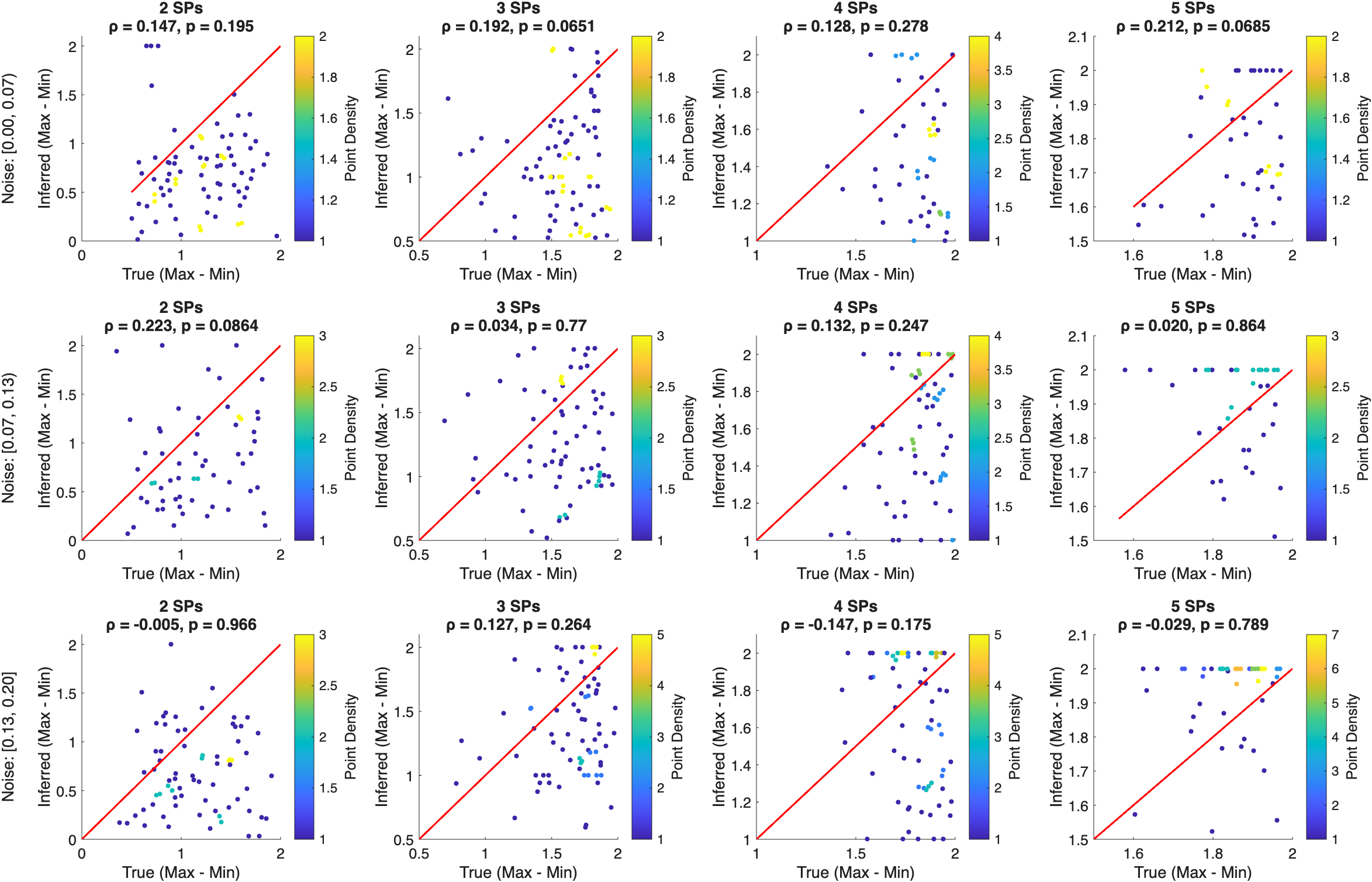

Supplement: S3 Fig — A grid of scatter plots illustrating the relationship between the true range (maximum - minimum) of the data and the inferred range, decomposed by the number of SPs and the level of noise (Ω). Each panel shows the density of data points, with warmer colors indicating a higher concentration of simulations falling within that region. The red line in each subplot represents the line of perfect agreement (y=x). Within each subplot, the Spearman rank correlation coefficient (ρ) and its corresponding p-value (p) are displayed, quantifying the linear relationship between the true and inferred ranges under that specific condition. (TIFF) [file pcbi.1013897.s004.tiff]

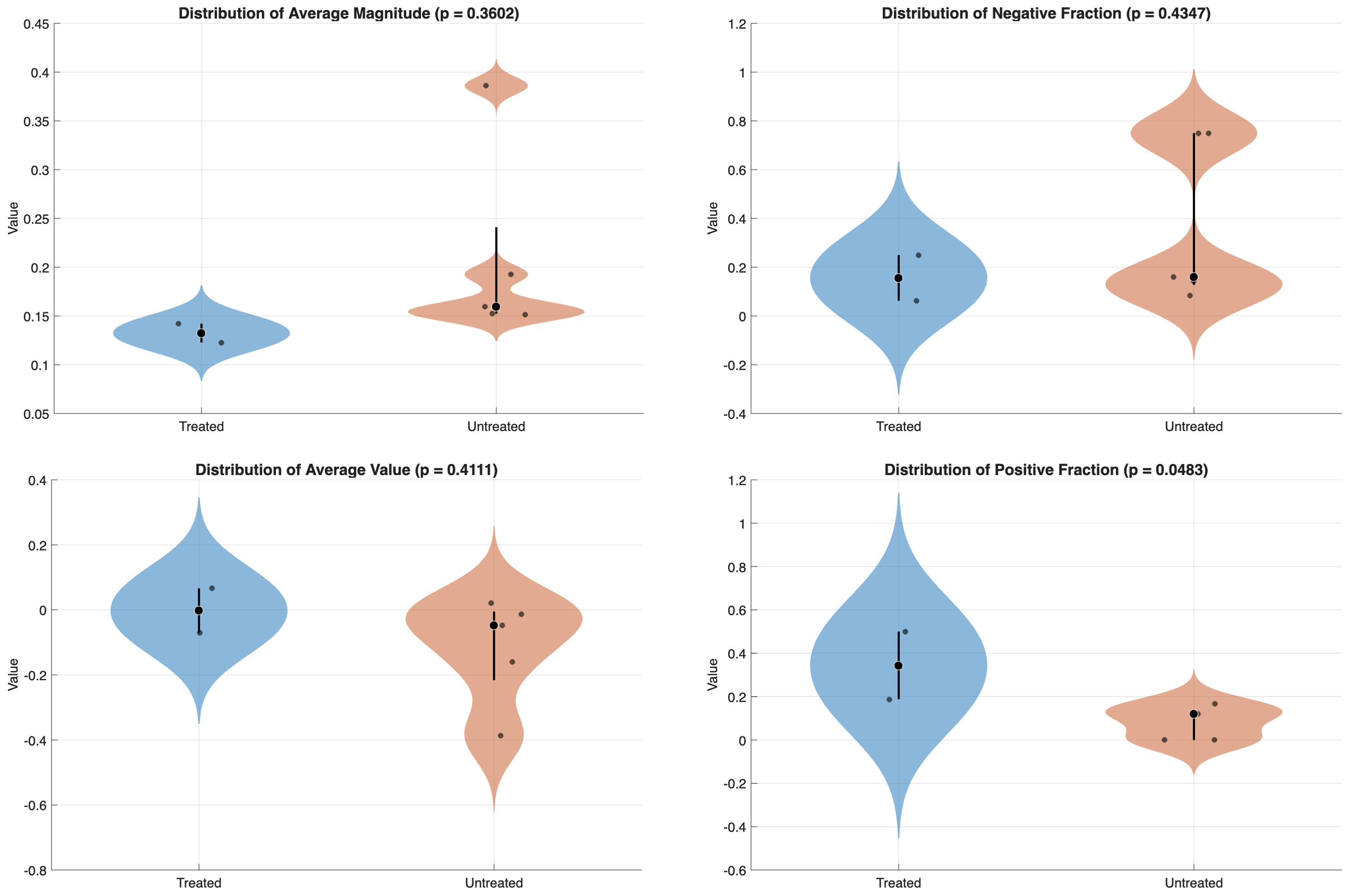

Supplement: S4 Fig — The distribution of four key metrics between treated and untreated sample groups: average magnitude, average value, positive fraction, and negative fraction of payoff matrix entries. Violin plots illustrate the data distribution for each group, with individual data points shown as black dots. A black line represents the interquartile range (IQR), and a white dot with a black outline indicates the median. P-values from an independent samples t-test are included in the title of each subplot to indicate the statistical significance of the difference between the groups for each metric. (TIFF) [file pcbi.1013897.s005.tiff]

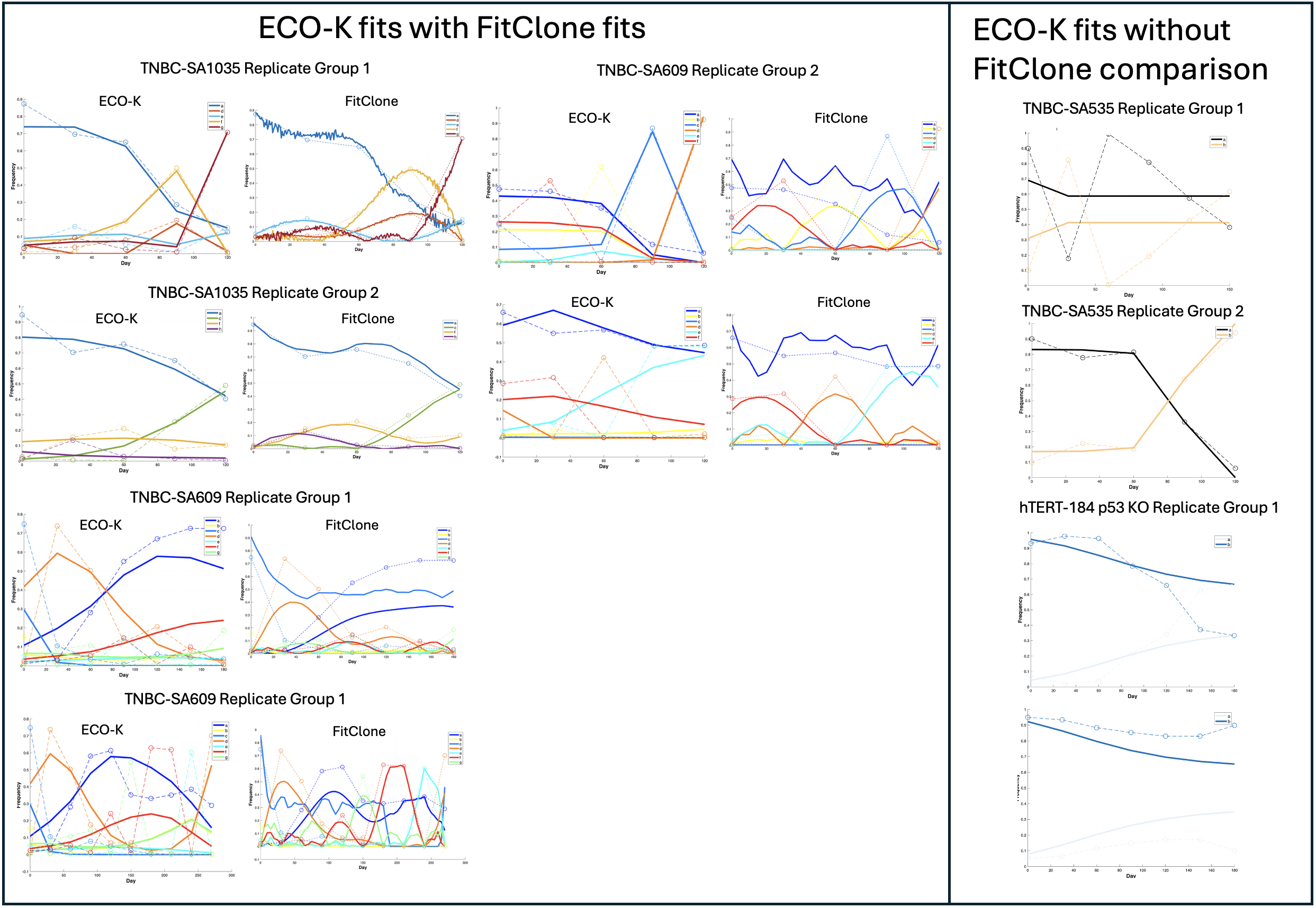

Supplement: S5 Fig — ECO-K fits are shown alongside corresponding FitClone fits (left panels) for replicate groups with three or more subpopulations (SPs), including TNBC-SA1035 (Replicate Groups 1–2) and TNBC-SA609 (Replicate Groups 1–2). For replicate groups with only two SPs (TNBC-SA535 Replicate Groups 1–2 and hTERT-184 p53 KO Replicate Group 1), only ECO-K fits are shown, as FitClone requires more than two SPs for inference. This comparison highlights the consistency and differences in inferred SP dynamics between the two approaches. (TIFF) [file pcbi.1013897.s006.tiff]

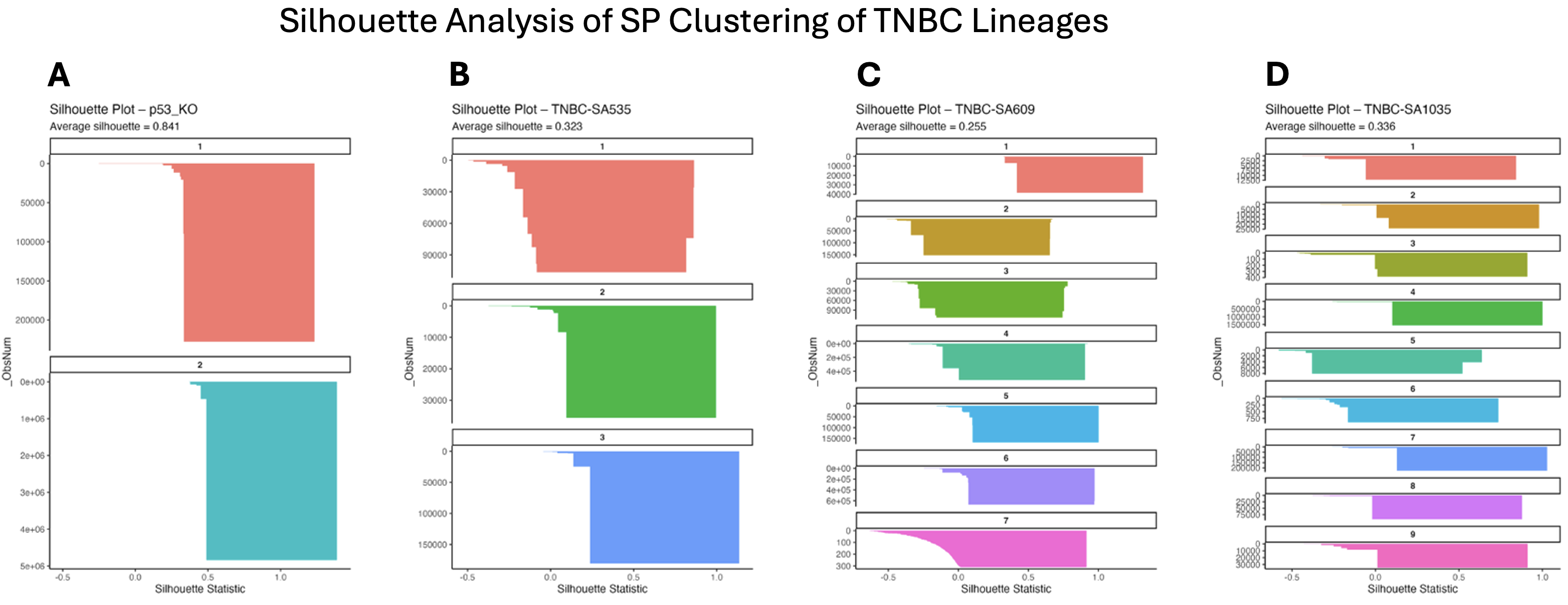

Supplement: S6 Fig — (A) For the hTERT-184 p53 KO cell line, the total within-cluster sum of squares was identical, and the mean silhouette was 0.841 (CV = 0%). All 25 runs converged to the same optimum, indicating a uniquely stable clustering for this dataset. (B) For TNBC-SA535, the total WCSS varied by 4.7%, and the mean silhouette width varied by 0.058 (CV = 20%). 18 of 25 runs converged to two high-quality, closely related optima. (C) TNBC-SA609, the WCSS varied by only 2.3%, and the mean silhouette width varied by 0.027 (CV = 12%). 17 of 25 runs converged to two nearly identical optima. (D) For TNBC-SA1035, the total WCSS varied by 4.7%, and the mean silhouette width varied by 0.031 (CV = 9.8%). 22 of 25 runs converged to two nearly identical optima. (TIFF) [file pcbi.1013897.s007.tiff]

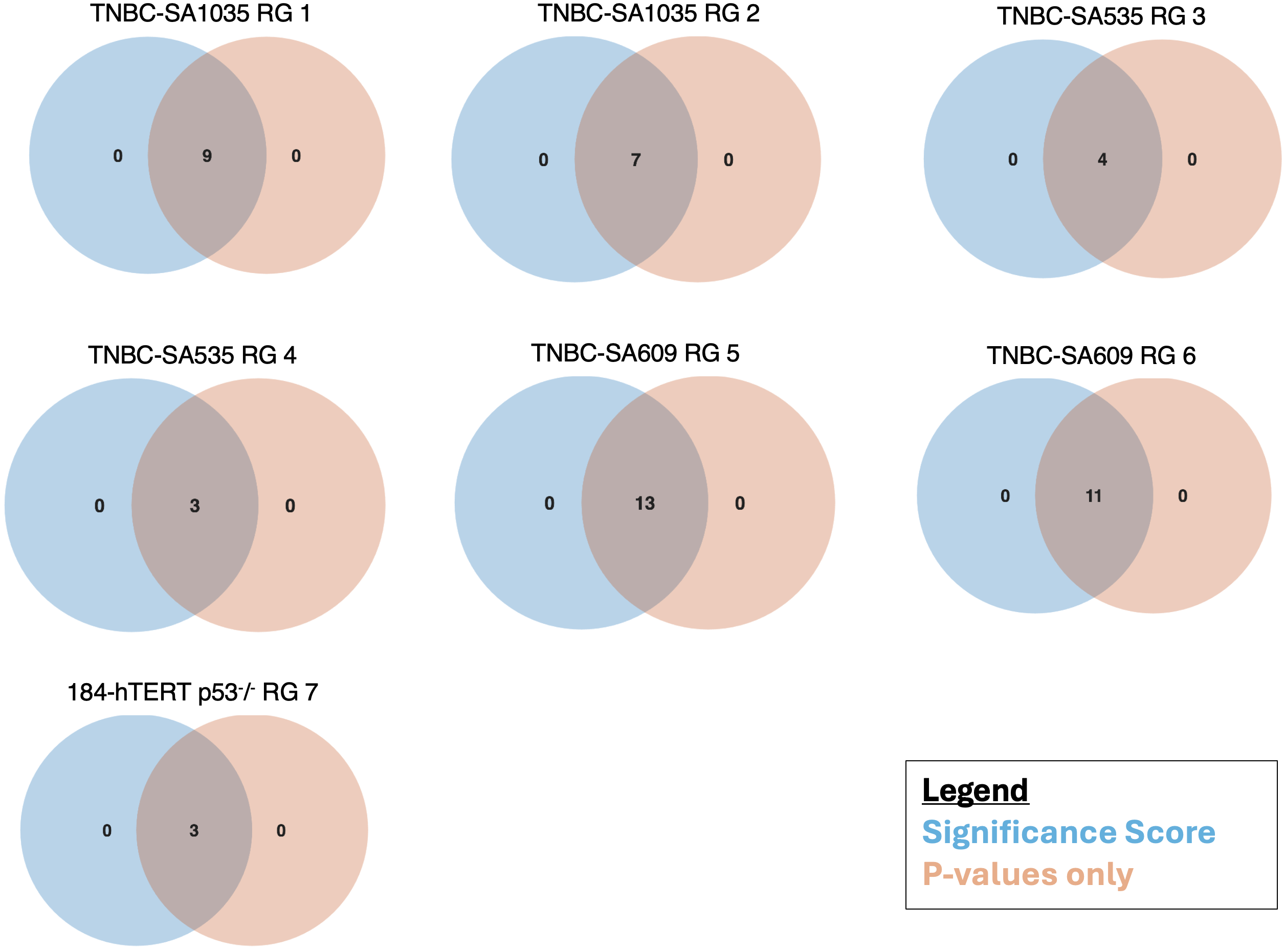

Supplement: S7 Fig — Analyses of SP frequency data from in-vitro evolution experiments to find significant growth interactions between different SPs. A two sample t-test (MATLAB R2025a “ttest2” function) was used to calculate interaction strengths and p-values, followed by two distinct methods to select the most significant interactions: one based on a composite significance score (Methods Sect 2.3), the other on p-values alone. (TIFF) [file pcbi.1013897.s008.tiff]

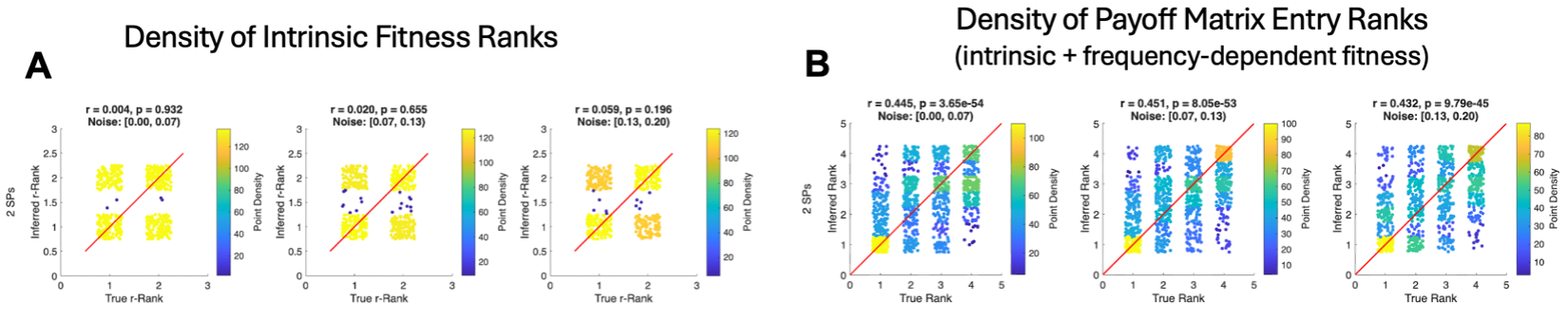

Supplement: S8 Fig — (A) Recovery of intrinsic fitness ranks under the hybrid model for synthetic 2-SP systems across increasing noise levels. In all cases, the model fails to recover the true intrinsic fitness order (Spearman’s ρ ≈ 0; p > 0.05), demonstrating that baseline fitness cannot be reliably inferred from relative-frequency trajectories alone. (B) Recovery of payoff matrix entry ranks when both intrinsic fitness and frequency-dependent effects are present. Although payoff inference remains partially informative (ρ 0.43–0.45), performance is degraded compared to the frequency-dependent-only setting (Fig 2B in main text). Across noise regimes, the hybrid model systematically fails to disentangle intrinsic fitness from payoff contributions, resulting in lower rank accuracy. (TIFF) [file pcbi.1013897.s009.tiff]
